# Prediction of 316 stainless steel low-cycle fatigue life based on machine learning

**DOI:** 10.1038/s41598-023-33354-1

**Published:** 2023-04-25

**Authors:** Hongyan Duan, Mengjie Cao, Lin Liu, Shunqiang Yue, Hong He, Yingjian Zhao, Zengwang Zhang, Yang liu

**Affiliations:** 1 School of Mechanical and Electrical Engineering, Lanzhou University of Technology, Lanzhou, 730050 China; 2 Wenzhou Engineering Institute of Pump&Valve, Lanzhou University of Technology, Lanzhou, China

**Keywords:** Mechanical engineering, Mechanical properties, Metals and alloys

## Abstract

The low-cycle fatigue life of 316 stainless steel is a significant basis for safety assessment. Usually, many factors affect the low-cycle fatigue life of stainless steel, and the relationship between the influencing factors and fatigue life is complicated and nonlinear. Therefore, it is hard to predict fatigue life using the traditional empirical formula. Based on this, a machine learning algorithm is proposed. In this paper, based on the large amount of existing experimental data, machine learning methods are used to predict the low circumferential fatigue life of 316 stainless steel. The results show that the prediction accuracy of nu-SVR and ELM models is high and can meet engineering needs.

## Introduction

316 stainless steel is a widely used type of chromium-nickel stainless steel. It is commonly used in food processing, medical equipment, the nuclear industry, chemical production, and other fields with harsh and strict requirements because of its good high-temperature fatigue performance, toughness, and corrosion resistance. Given the increasingly complex working conditions of 316 stainless steel, its safety is a top priority for consideration in engineering applications, and fatigue life failure is an important basis for safety assessments^1,2^. It is important to study the prediction of low-cycle fatigue life. The model most often used for the low-cycle fatigue life prediction of 316 stainless steel is the traditional empirical formula prediction method. The main models are cumulative damage theory^3^, local stress–strain^4^, energy method^5^, and field strength method^6^. In traditional fatigue life prediction, the relationship between fatigue life and influencing factors is determined based on a large number of experiments, and the fatigue life is predicted by applying a large number of empirical formulas. The traditional empirical formula fatigue life prediction model has severe limitations, such as the variety of empirical formulas, low prediction accuracy, high and repeated experimental costs, and long prediction time; the development of machine learning has provided new ideas to solve these problems^7–16^.

Machine learning (ML) is a multi-disciplinary field that incorporates theories from a variety of disciplines, which include probability theory, statistics, approximation theory, convex analysis, algorithmic complexity, etc.^17^. In simple terms, machine learning is a way of learning through computer simulation of human learning, where machine learning continuously trains models from data, thereby improving their generalization^18^. Due to the powerful abilities of machine learning such as data processing and data analysis, the method has been widely used in the fields of data mining, automatic speech recognition, computer vision, and fault detection and diagnosis. At present, it also has some applications in life prediction^19–22^. However, there are few studies on low-cycle fatigue life prediction of 316 stainless steel using a machine learning model.

In this paper, the low-cycle fatigue life of 316 stainless steel is predicted by machine learning. Firstly, based on the collected literature data, the effects of factors such as stress intensity factor, strain amplitude, and residual stress on the low-cycle fatigue life of 316 stainless steel are summarized. Secondly, a sensitivity analysis and pre-processing of the collected data were carried out to ensure a prediction model with less error. Finally, machine learning models such as BP neural network, genetic algorithm optimized BP neural network, limit learning machine, and support vector machine were established to predict the low-cycle fatigue life of 316 austenitic stainless steel.

## Effects of different factors on low-cycle fatigue life of 316 stainless steel

### Effect of stress intensity factor

**Figure 1:**
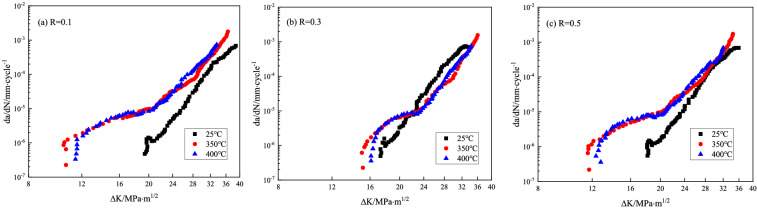
The stress intensity factor affects the crack growth rate at different temperatures under (**a**) R = 0.1, (**b**) R = 0.3, (**c**) R = 0.5.

Figure1^23–28^shows the influence of the stress intensity factor on the crack growth rate under different temperatures and stress ratios. As can be seen from the figure, no matter whether the stress ratio is 0.1, 0.3, or 0.5, the crack growth rate increases with an increase in the stress intensity factor at the same temperature, but the increase rate varies with the temperature.

### Effect of strain amplitude

**Figure 2:**
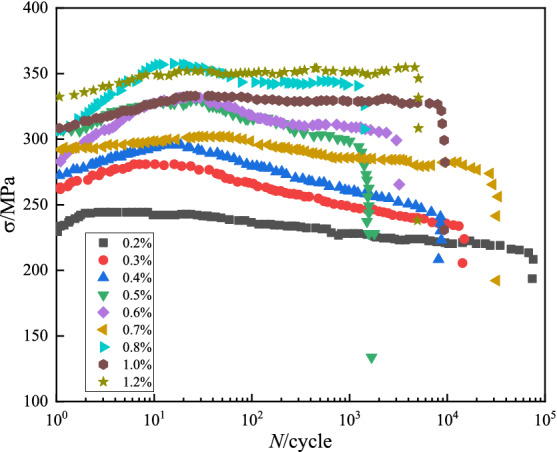
The relationship between stress and cyclic cycles under different strain amplitudes.

Figure2^29–33^shows typical cyclic stress response curves for different strain amplitudes. It can be seen that the cyclic characterization of the material is correlated with the strain amplitude. At a low strain amplitude (0.2%), the material does not show hardening, and the cycles are longer than other strain amplitudes. As the strain amplitude increases (before 0.8%), the material stress cycle response exhibits two phases. However, at high strain amplitude, the cyclic response of the material exhibits three phases. When the strain amplitude is 0.5%, the stress drops sharply and the number of cycles is the lowest. It can be seen from the figures that, as the strain amplitude increases from 0.2 to 1.2%, the cycle time gradually decreases from${10}^{5}$to${10}^{3}$. The distribution of all data is exponential, which is typical of the ε-N (strain-cycle) distribution of the low-cycle fatigue life of stainless steel.

### Effect of residual stress

Residual stresses are the mutually balanced internal stresses that exist within the material or part when no external forces are applied. Residual stress includes compressive residual stress and tensile residual stress. Compressive residual stress is beneficial to materials and can effectively inhibit crack propagation, while tensile residual stress is harmful to materials and should be eliminated as far as possible. If the surface treatment of 316 stainless steel is carried out, the residual compressive stress can be increased. If the surface treatment of 316 stainless steel is continued, the residual compressive stress will cause stress relaxation under cyclic load, resulting in the reduction or even disappearance of the effect of residual compressive stress on increasing the fatigue life of materials^34^.

## Data processing

### Sensitivity analysis based on the Sobol algorithm

In this paper, the low-cycle fatigue life prediction of 316 stainless steel is studied. The three factors considered above, crack growth rate, average strain, and residual stress, were taken as the input data of machine learning, and the fatigue life was taken as the output data to establish a machine learning prediction model. The total number of samples was 500 groups^23–50^. Due to the large amount of literature data, only a few studies are listed in the references.

The Sobol^51^method was used to study the effect of different input variables on the low perimeter fatigue life of 316 stainless steel. The core of the Sobol algorithm is to decompose the total variance of the objective function into the variance of a single objective and multiple objective parameters.

Let the model be expressed as$u=f\left(x\right)$, where the model parameters$x = x_{1} ,x_{2} , \ldots ,x_{n}$are n-dimensional discrete points and u is the output^52^.

If the function$f\left(x\right)$be productive and${x}_{i}$obey uniform distribution in$\left[0,1\right]$, then$f\left(x\right)$can be expressed as follows:

1 $$f\left( x \right) = f_{0} + \sum\limits_{s = 1}^{n} {\sum\limits_{{i_{1} < \cdot \cdot \cdot < i_{s} }}^{n} {f_{{i_{1} \cdot \cdot \cdot i_{s} }} } } \left( {x_{i} , \cdot \cdot \cdot ,x_{{i_{s} }} } \right),$$

where$1\le {i}_{1}\ldots <{i}_{s}\le n \left(1\le s\le n\right)$, there are${2}^{n}$terms in the summed number. Equation (1) is the expression of variance decomposition of the function$f\left(x\right)$.

The total variance of the model can also be decomposed as a combination between one parameter and several other parameters:

2 $$Var\left( Y \right) = \sum\limits_{i} {Var\left( Y \right)_{i} } + \sum\limits_{i < j} {Var\left( Y \right)_{ij} } + \cdot \cdot \cdot + Var\left( Y \right)_{1,2, \ldots ,n} ,$$

where*Var(Y)*is the total variance of the model;${Var(Y)}_{i}$is the variance generated by a parameter${x}_{i}$;${Var(Y)}_{ij}$is the variance generated by the interaction of parameters${x}_{i}$and${x}_{j}$; and${Var(Y)}_{\mathrm{1,2},\cdots ,n}$is the variance generated by the joint action of*n*parameters. Normalizing the above equation, the sensitivity between the parameters is obtained as follows:

3 $$1 = \sum\limits_{i} {\frac{{Var\left( Y \right)_{i} }}{Var\left( Y \right)}} + \sum\limits_{i < j} {\frac{{Var\left( Y \right)_{ij} }}{Var\left( Y \right)}} + \cdot \cdot \cdot + \frac{{Var\left( Y \right)_{1,2, \ldots ,n} }}{Var\left( Y \right)}.$$

Then, the full-order sensitivity of the model can be expressed as follows:

- First-order sensitivity index:${S}_{i}=\frac{{Var(Y)}_{i}}{Var(Y)}$;
- Second-order sensitivity index:${S}_{ij}=\frac{{Var(Y)}_{ij}}{Var(Y)}$;
- Total sensitivity index:${S}_{Ti}=1-\frac{{Var(Y)}_{\sim i}}{Var(Y)}$.

### Data normalization

Due to the different types of data collected and the different magnitudes of the data, they are often discrete. If network input is performed, it will cause the annihilation of the data and a loss of information. For better generalization, the collected data were normalized by the following formula^53^:

4 $$x^{\prime} = \frac{{2\left( {x - x_{\min } } \right)}}{{x_{\max } - x_{\min } }} - 1,$$

where$x$and${x}^{\mathrm{^{\prime}}}$are the values before and after data normalization, respectively;${x}_{min}$and${x}_{max}$are the minimum and maximum values inside the collected sample data, respectively.

The randperm function was used to disrupt the sample order, the total number of samples was 500 groups, 450 groups were randomly selected as training data, and 50 groups were used as test data.

## Machine learning model

### BP neural network and genetic algorithm to optimize BP neural network model

BP (back propagation) neural network is the most basic neural network; its output results are forward-propagated, and the error is backpropagated. The neural network power threshold is adjusted according to the prediction error. The basic unit of a neural network is the neuron, and the basic architecture is composed of the input layer, the hidden layer, and the output layer. According to Kolmogorov's theorem, a three-layer BP neural network structure has a solid nonlinear mapping capability and can approximate any nonlinear function^54^.

**Figure 3:**
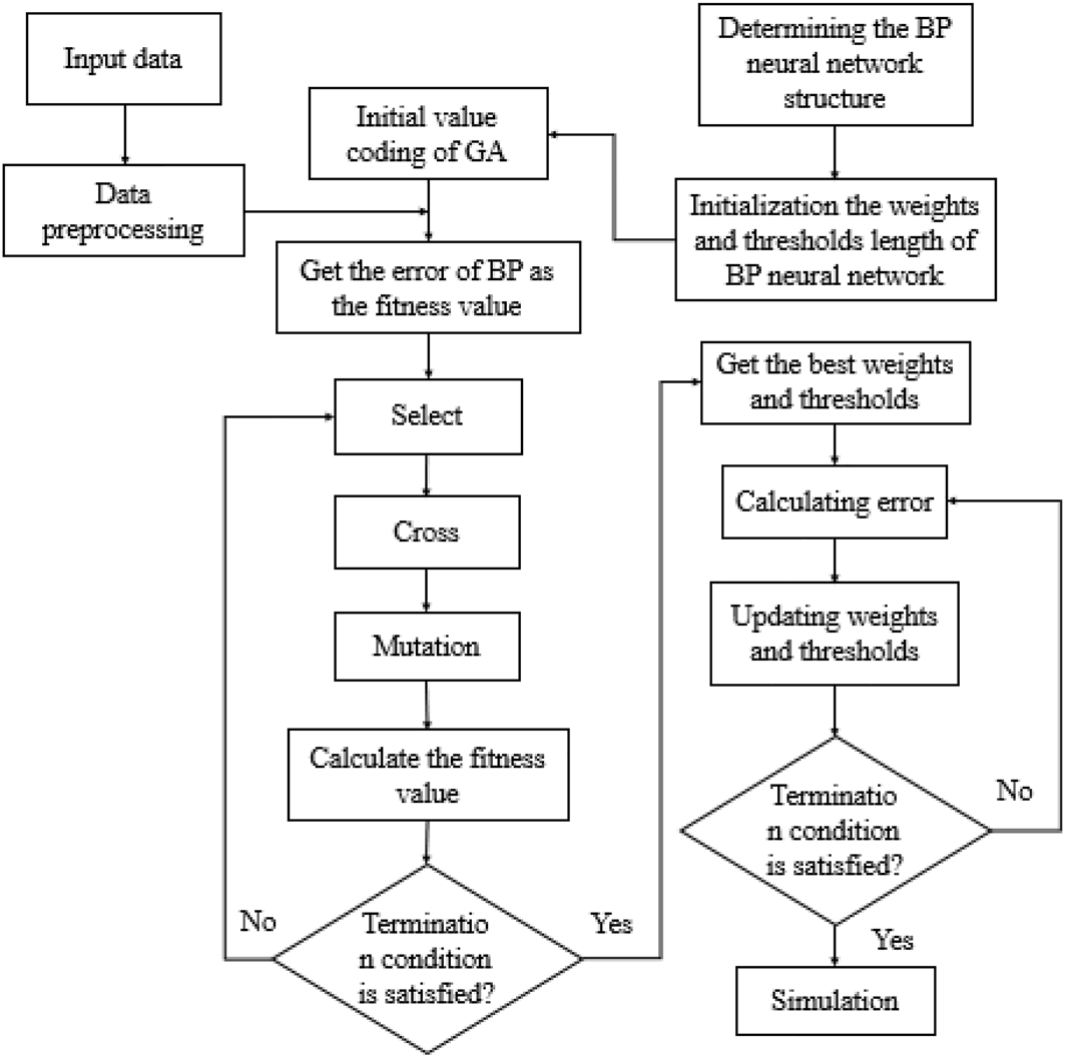
Genetic algorithm optimizes the BP neural network process.

Since the BP neural network uses the fastest gradient descent method to learn an artificial neural network, and the initial weights and thresholds of the BP neural network are randomly generated, it is easy to fall into the optimal local solution during the training of the BP neural network, which makes the prediction error large and the generalization ability of the model not strong. A genetic algorithm (GA) is mainly an algorithm for global search and optimization based on simulating the biological evolution mechanism in nature, which in turn can solve the BP neural network in the case of local optimal conditions^55–58^.The genetic algorithm optimizes the connection weight and threshold of BP neural network. The entire process is shown in Fig.3.

### Extreme learning machine

Extreme Learning Machine (ELM) is a new Single-hidden-Layer Feedforward Neural Network (SLFN) learning algorithm^59,60^.ELM adjusts the number of neurons in the hidden layer without adjusting other weight thresholds and the hidden layer. Compared with other algorithmic models, ELM has the advantages of fast training and good generalization performance. It is now widely used in the fields of life prediction, reliability, and fault diagnosis.

Two theorems were proposed by Huang et al.^59^:

#### Theorem 1

Given any Q distinct samples$\left({x}_{i},{t}_{i}\right)$, where${x}_{i}={\left[{x}_{i1},{x}_{i2},\dots {x}_{im}\right]}^{T}\in {R}^{n},{t}_{i}=\left[{t}_{i1},{t}_{i2},\dots {t}_{im}\right]\in {R}^{m}$and an arbitrary interval infinitely differentiable activation function*g*:$R\to R$, then for a SLFN with Q hidden layer neurons, with any assignment${w}_{i}\in {R}^{n}$and${b}_{i}\in R$, its hidden layer output matrix*H*is invertible and has$\Vert H\beta -{T}^{^{\prime}}\Vert =0$.

#### Theorem 2

Given any Q distinct samples$\left({x}_{i},{t}_{i}\right)$, where${x}_{i}={\left[{x}_{i1},{x}_{i2},\dots {x}_{im}\right]}^{T}\in {R}^{n},{t}_{i}=\left[{t}_{i1},{t}_{i2},\dots {t}_{im}\right]\in {R}^{m}$, and given any small error$\varepsilon >0$, and an arbitrary interval infinitely differentiable activation function*g*:$R\to R$, there always exists a SLFN containing*K*(*K*≤*Q*) hidden layer neurons with$\Vert {H}_{N\times M}{\beta }_{M\times m}-{T}^{^{\prime}}\Vert <\varepsilon$for any assignment${w}_{i}\in {R}^{n}$and${b}_{i}\in R$.

The weights and biases are randomly generated before ELM training, so only the number of hidden layer neurons and the activation function must be determined to calculate β. The steps are as follows:

1. Determine the number of neurons in the hidden layer and set the weights*w*and bias*b*.
2. Set the activation function as an infinitely differentiable function, and then calculate the output matrix*H*of the hidden layer.
3. Calculate the output layer weights$\beta :\widehat{\beta }={H}^{+}{T}^{^{\prime}}$.

### Support vector machine

**Figure 4:**
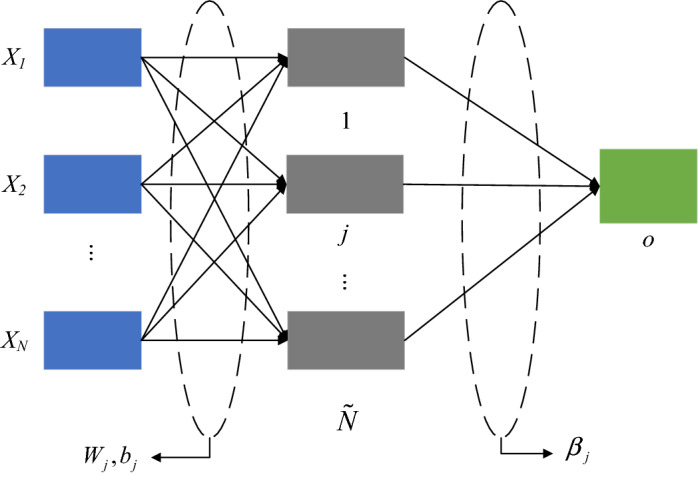
SVM system structure.

Support Vector Machine (SVM) is often used in classification and nonlinear regression problems. The main idea is to find the maximum geometric distance by controlling the function distance, which means that the function distance is the constraint and the geometric distance is the objective function. The SVM algorithm architecture is shown in Fig.4:where*K*is the kernel function, and its main types are as follows:

- Linear kernel function:$k\left( {x_{i} ,x_{j} } \right) = x_{i}^{T} x_{j}$;
  Polynomial kernel function:$k\left( {x_{i} ,x_{j} } \right) = \left( {x_{i}^{T} x_{j} } \right)^{d}$;
  Gaussian kernel function:$k\left( {x_{i} ,x_{j} } \right) = \exp \left( { - \frac{{||x_{i} - x_{j} ||^{2} }}{{2\sigma^{2} }}} \right)$;
  The Laplace kernel function:$k\left( {x_{i} ,x_{j} } \right) = \exp \left( { - \frac{{||x_{i} - x_{j} ||}}{\sigma }} \right)$;
  Sigmoid kernel function:$k\left( {x_{i} ,x_{j} } \right) = \tanh \left( {\beta x_{i}^{T} x_{j} + \theta } \right)$.

## Results and analysis

### BP neural network and genetic algorithm to optimize BP neural network model

**Figure 5:**
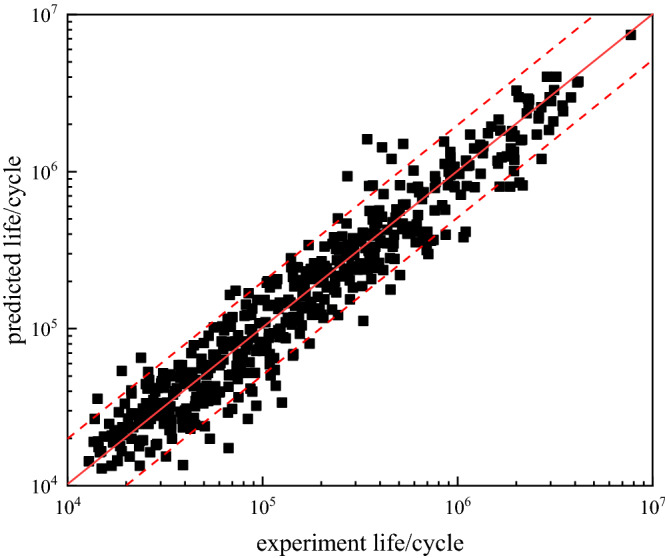
BP neural network training results.

Parameters such as the number of hidden layer neurons, activation function type, and backpropagation algorithm affect the BP neural network prediction performance.The parameters of the BP neural network are controlled, 1–20 dimensional neurons are selected, the tansig function and the logsin function are compared, and the tansig function has higher prediction accuracy than the logsin function. The LM (Levenberg–Marquardt) backpropagation algorithm, GD (Gradient descent) algorithm, and GDA (Gradient descent with adaptive learning rate) algorithm are compared. The LM algorithm is found to have higher prediction accuracy. When the optimal parameter neuron is determined to be 10, the tansig function is selected for the hidden layer function, and the LM algorithm is chosen. The prediction result is shown in Fig.5, and it can be seen that the predicted value is basically within the error band of twice.

**Figure 6:**
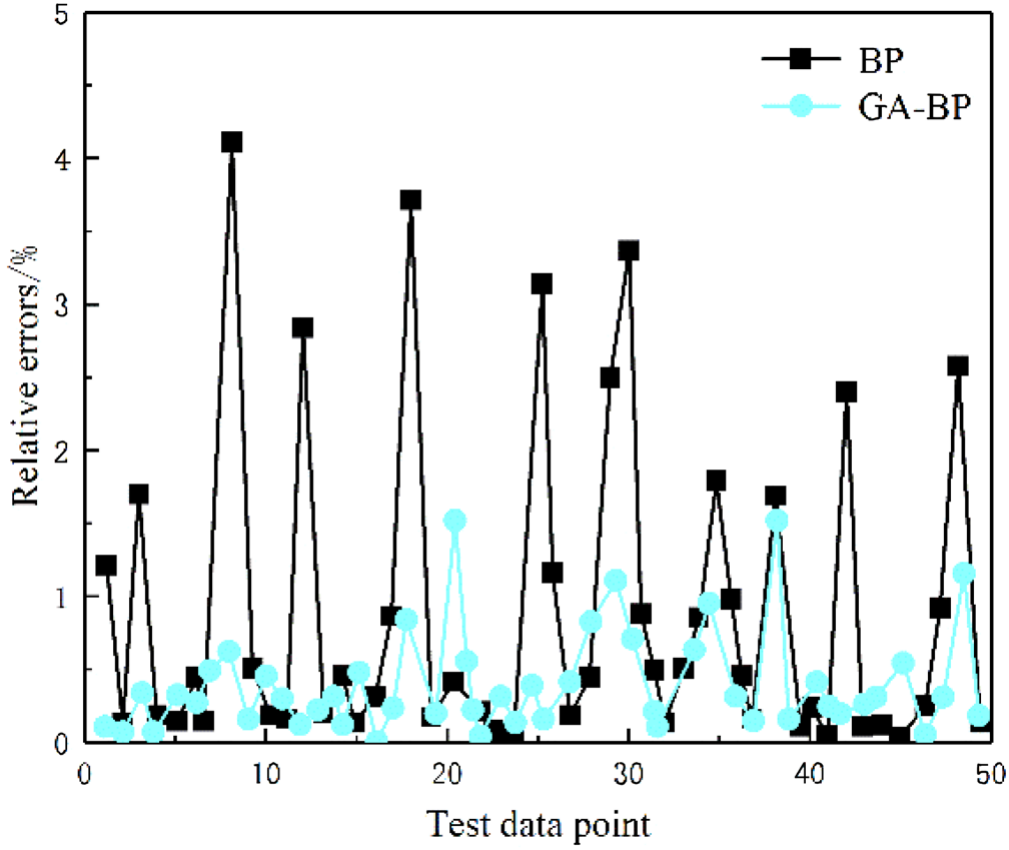
The prediction errors of the two models’ test sample.

The parameters of the genetic algorithm were set as follows: maxgen = 100, sizepop = 30, pcross = 0.3, and pmutation = 0.1. The prediction errors of the two models test sample (50 groups) are shown in Fig.6, which shows that the BP neural network fluctuates the most. The GA-BP neural network fluctuates within 2% relative error, and the training effect is more suitable than the BP neural network.

### Extreme learning machine

**Figure 7:**
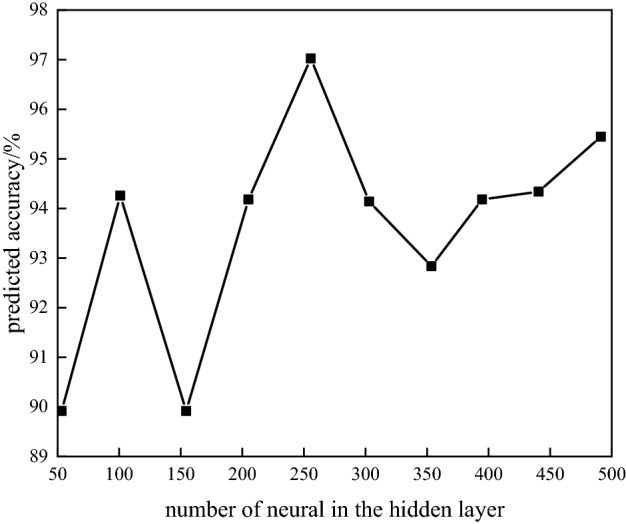
The influence of the number of hidden layer neurons on the performance of ELM.

In ELM prediction, the correct selection of parameters is crucial to the prediction results. The parameter selection of ELM mainly includes the selection of input and internal parameters. The input parameters are mainly the selection of data volume, and the internal parameters are the key factors affecting the prediction ability of ELM. The internal parameters are mainly the activation function and the number of neurons in the hidden layer; relatively speaking, the effect of the activation function on ELM is smaller than the effect of the number of neurons in the hidden layer. According to Theorems 1 and 2, the more neurons there are in the hidden layer, the more likely SLFN is to approximate all the training samples with zero error, and the better the results obtained by ELM prediction. However, when the number of neurons in the hidden layer is large enough, it will affect the generalization performance of ELM. As shown in Fig.7, the accuracy of the test set shows that the accuracy peaks at a specific value as the number of hidden layer neurons increases, and the accuracy of the training set will decrease if the number of hidden layer neurons continues to increase. Therefore, choosing the appropriate number of hidden layer neurons is necessary to achieve the optimal prediction accuracy of ELM.

### Support vector machine

**Figure 8:**
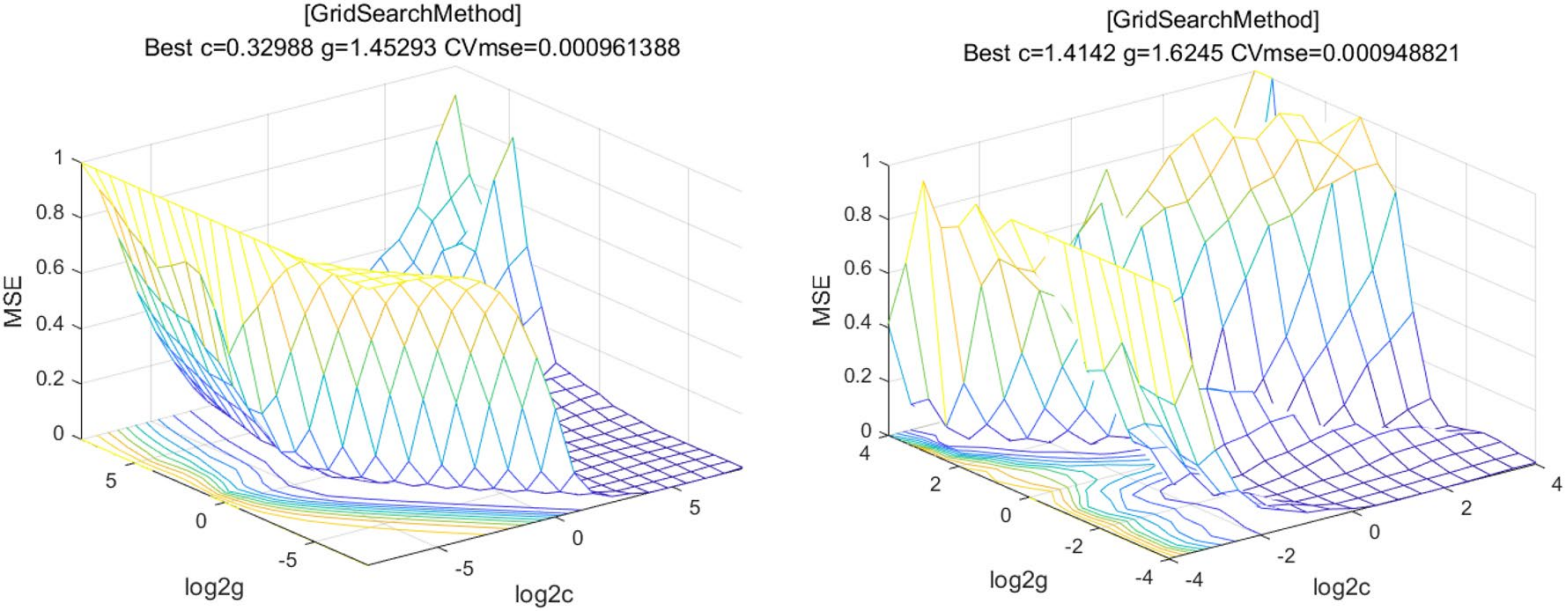
Result of parameter selection (rough selection diagram vs. fine selection map).

Regression analysis of the low perimeter fatigue life of 316 stainless steel wasperformed using the support vector machine toolbox LIBSVM developed in the literature^49^. Two regression support vector machine models (epsilon-SVR and nu-SVR) were selected for life prediction, both of which were chosen with Gaussian radial basis kernel functions. In solving the problem with SVM, the selection of parameters significantly impacts the SVM prediction. For the above two regression models, there are penalty coefficients*C*and kernel function parameters*g*. The cross-validation (CV) method can find parameters*C*and*g*, and the*C*and*g*obtained can avoid under-learning and over-learning states and finally achieve superior accuracy of data set prediction. As shown in Fig.8, the penalty coefficient*C*inside the rough selection is small, and the fine selection mean square error (MSE) has a smaller error than the rough selection. The best parameters are set as follows: penalty coefficient*C*= 1.4142, kernel function parameter*g*= 1.6245, and insensitivity coefficient*p*= 0.01. These parameters are used to construct the support vector machine prediction model.

**Figure 9:**
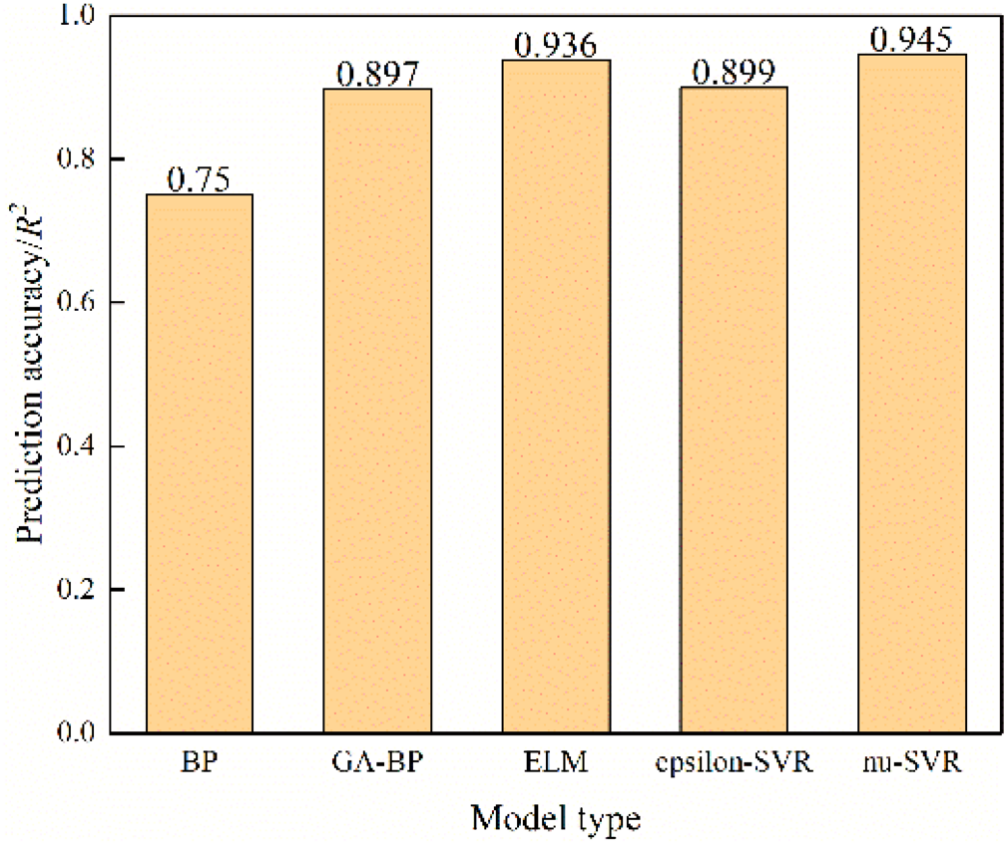
Model prediction accuracy.

The prediction accuracy*R*^*2*^of several models is shown in Fig.9. It can be seen that the BP prediction model has a poor effect, while the nu-SVR prediction model has the best effect.

## Conclusions

1. This paper discusses three factors that affect the fatigue life of 316 stainless steel. The influence of the stress intensity factor on crack rate under different temperatures and stress ratios is discussed in the first factor. The second factor compares the relationship between strain amplitude and cycle times. The third factor discusses the relationship between loading stress, stress ratio, cycle times, and residual stress.
2. To address the problem of large error between the traditional material fatigue life calculation method and the actual value, a low-cycle fatigue life prediction model of 316 stainless steel based on machine learning was established in this paper. The model took crack growth rate, average stress, and residual stress as input data and fatigue life as output data.
3. Compared with the centralized model proposed in this paper, the prediction effect of the BP neural network was poor. The prediction effect of the nu-SVR model was the best, followed by ELM, and the*R*^*2*^ reached 0.945 and 0.936, respectively, which met the project’s needs.

## Supplementary Information

Supplementary Information 1.

Supplementary Information 2.
