# Supplementary material for: Prediction of 316 stainless steel low-cycle fatigue life based on machine learning
Source: Sci Rep. 2023 Apr 25;13:6753. doi: 10.1038/s41598-023-33354-1 (PMC10130168; doi:10.1038/s41598-023-33354-1)
Supplement: Supplementary file 1 — Supplementary Information 1. [file 41598_2023_33354_MOESM1_ESM.docx]

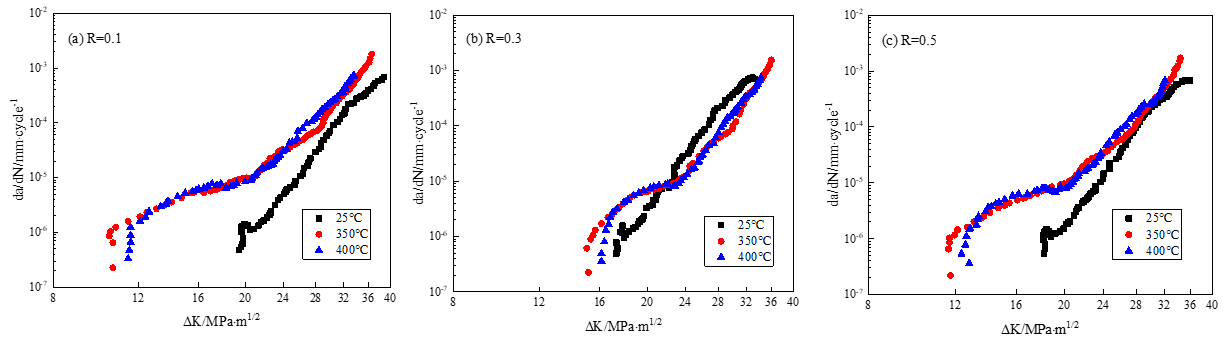


**Fig. 1.** The stress intensity factor affects the crack growth rate at different temperatures under (a) R = 0.1, (b) R= 0.3, (c) R = 0.5.

**Table. 1(a).** Fig.1 (a)’s data

| Temperature, ℃ | da/dN, mm/N | $\Delta$K |
| --- | --- | --- |
| 25 | 4.47E-07 | 25.71215 |
| 25 | 5.21E-07 | 25.90387 |
| 25 | 5.95E-07 | 26.08898 |
| 25 | 6.58E-07 | 26.05592 |
| 25 | 7.39E-07 | 26.12864 |
| 25 | 8.76E-07 | 26.01626 |
| 25 | 9.84E-07 | 25.93692 |
| 25 | 1.09E-06 | 25.90387 |
| 25 | 1.27E-06 | 25.97659 |
| 25 | 1.40E-06 | 26.08898 |
| 25 | 1.43E-06 | 26.24103 |
| 25 | 1.35E-06 | 26.54513 |
| 25 | 1.29E-06 | 26.66413 |
| 25 | 1.17E-06 | 26.77651 |
| 25 | 1.11E-06 | 26.92857 |
| 25 | 1.07E-06 | 27.193 |
| 25 | 1.25E-06 | 27.64916 |
| 25 | 1.47E-06 | 27.9136 |
| 25 | 1.75E-06 | 28.18465 |
| 25 | 1.87E-06 | 28.48875 |
| 25 | 2.06E-06 | 28.86557 |
| 25 | 2.52E-06 | 28.79285 |
| 25 | 2.99E-06 | 29.20934 |
| 25 | 3.59E-06 | 29.36139 |
| 25 | 4.04E-06 | 29.62583 |
| 25 | 4.94E-06 | 29.85721 |
| 25 | 4.79E-06 | 30.12165 |
| 25 | 5.75E-06 | 30.23404 |
| 25 | 6.70E-06 | 30.57781 |
| 25 | 7.04E-06 | 30.9943 |
| 25 | 8.20E-06 | 30.96124 |
| 25 | 8.77E-06 | 31.07363 |
| 25 | 1.04E-05 | 31.6025 |
| 25 | 1.21E-05 | 31.87355 |
| 25 | 1.48E-05 | 31.83389 |
| 25 | 1.66E-05 | 32.29004 |
| 25 | 1.93E-05 | 32.32971 |
| 25 | 2.29E-05 | 32.51481 |
| 25 | 2.84E-05 | 33.01063 |
| 25 | 3.36E-05 | 33.39407 |
| 25 | 3.85E-05 | 33.50646 |
| 25 | 4.40E-05 | 33.54612 |
| 25 | 4.63E-05 | 33.81056 |
| 25 | 5.12E-05 | 33.92294 |
| 25 | 6.05E-05 | 34.26671 |
| 25 | 6.36E-05 | 34.26671 |
| 25 | 7.54E-05 | 34.33943 |
| 25 | 8.20E-05 | 34.57082 |
| 25 | 9.22E-05 | 34.83525 |
| 25 | 1.11E-04 | 35.29141 |
| 25 | 1.25E-04 | 35.33108 |
| 25 | 1.38E-04 | 35.63518 |
| 25 | 1.55E-04 | 35.74757 |
| 25 | 1.69E-04 | 35.93928 |
| 25 | 1.93E-04 | 35.93928 |
| 25 | 2.21E-04 | 36.05167 |
| 25 | 2.13E-04 | 36.39544 |
| 25 | 2.13E-04 | 36.73921 |
| 25 | 2.61E-04 | 36.77887 |
| 25 | 2.84E-04 | 37.11603 |
| 25 | 2.99E-04 | 37.53913 |
| 25 | 3.19E-04 | 37.69118 |
| 25 | 3.54E-04 | 37.91595 |
| 25 | 3.98E-04 | 38.10767 |
| 25 | 4.41E-04 | 38.25972 |
| 25 | 4.71E-04 | 38.52416 |
| 25 | 5.20E-04 | 38.67621 |
| 25 | 5.30E-04 | 38.86793 |
| 25 | 5.77E-04 | 39.0927 |
| 25 | 6.71E-04 | 39.43647 |
| 350 | 2.17E-07 | 13.77279 |
| 350 | 6.37E-07 | 13.77279 |
| 350 | 8.61E-07 | 13.46868 |
| 350 | 9.84E-07 | 13.50174 |
| 350 | 1.23E-06 | 13.99756 |
| 350 | 1.60E-06 | 15.14125 |
| 350 | 1.96E-06 | 16.27833 |
| 350 | 2.32E-06 | 16.88654 |
| 350 | 2.61E-06 | 17.91785 |
| 350 | 2.89E-06 | 18.67811 |
| 350 | 3.24E-06 | 18.83016 |
| 350 | 3.53E-06 | 19.31937 |
| 350 | 3.97E-06 | 20.35068 |
| 350 | 4.70E-06 | 20.4234 |
| 350 | 5.12E-06 | 20.80683 |
| 350 | 4.94E-06 | 21.22332 |
| 350 | 4.86E-06 | 21.41504 |
| 350 | 5.56E-06 | 21.52742 |
| 350 | 6.15E-06 | 21.91086 |
| 350 | 5.19E-06 | 22.32735 |
| 350 | 5.56E-06 | 22.7835 |
| 350 | 5.65E-06 | 23.04794 |
| 350 | 5.94E-06 | 23.43138 |
| 350 | 6.05E-06 | 23.76853 |
| 350 | 6.92E-06 | 24.19163 |
| 350 | 7.53E-06 | 24.72051 |
| 350 | 7.91E-06 | 24.91223 |
| 350 | 8.61E-06 | 25.06428 |
| 350 | 8.61E-06 | 25.44771 |
| 350 | 8.90E-06 | 25.63282 |
| 350 | 9.05E-06 | 25.93692 |
| 350 | 9.21E-06 | 26.32036 |
| 350 | 9.21E-06 | 26.47241 |
| 350 | 1.04E-05 | 27.04095 |
| 350 | 1.11E-05 | 27.42439 |
| 350 | 1.23E-05 | 27.57644 |
| 350 | 1.33E-05 | 27.72849 |
| 350 | 1.45E-05 | 27.84088 |
| 350 | 1.63E-05 | 28.14498 |
| 350 | 1.93E-05 | 28.60113 |
| 350 | 1.93E-05 | 28.60113 |
| 350 | 2.03E-05 | 28.75319 |
| 350 | 2.17E-05 | 29.01762 |
| 350 | 2.48E-05 | 29.13001 |
| 350 | 2.70E-05 | 29.40106 |
| 350 | 3.04E-05 | 29.9696 |
| 350 | 3.25E-05 | 30.38609 |
| 350 | 3.54E-05 | 30.57781 |
| 350 | 3.60E-05 | 30.92158 |
| 350 | 3.92E-05 | 31.07363 |
| 350 | 4.40E-05 | 31.64217 |
| 350 | 4.63E-05 | 31.75455 |
| 350 | 5.04E-05 | 31.94627 |
| 350 | 5.48E-05 | 32.05866 |
| 350 | 5.86E-05 | 32.44209 |
| 350 | 6.47E-05 | 32.63381 |
| 350 | 6.92E-05 | 32.78586 |
| 350 | 7.28E-05 | 32.97097 |
| 350 | 7.67E-05 | 33.46679 |
| 350 | 8.32E-05 | 33.46679 |
| 350 | 9.52E-05 | 33.65851 |
| 350 | 1.13E-04 | 33.81056 |
| 350 | 1.19E-04 | 33.85022 |
| 350 | 1.19E-04 | 33.85022 |
| 350 | 1.33E-04 | 34.00228 |
| 350 | 1.33E-04 | 34.00228 |
| 350 | 1.33E-04 | 34.00228 |
| 350 | 1.52E-04 | 34.22705 |
| 350 | 1.66E-04 | 34.41877 |
| 350 | 1.93E-04 | 34.53115 |
| 350 | 2.10E-04 | 34.79559 |
| 350 | 2.10E-04 | 34.79559 |
| 350 | 2.41E-04 | 35.09969 |
| 350 | 2.66E-04 | 35.29141 |
| 350 | 2.94E-04 | 35.55585 |
| 350 | 3.15E-04 | 35.63518 |
| 350 | 3.15E-04 | 35.63518 |
| 350 | 3.15E-04 | 35.63518 |
| 350 | 3.37E-04 | 35.63518 |
| 350 | 3.65E-04 | 35.8269 |
| 350 | 3.91E-04 | 36.012 |
| 350 | 4.55E-04 | 36.39544 |
| 350 | 4.95E-04 | 36.62682 |
| 350 | 5.39E-04 | 36.81193 |
| 350 | 5.95E-04 | 36.93092 |
| 350 | 6.93E-04 | 37.08298 |
| 350 | 7.54E-04 | 37.19536 |
| 350 | 8.48E-04 | 37.4598 |
| 350 | 9.39E-04 | 37.53913 |
| 350 | 9.87E-04 | 37.65152 |
| 350 | 0.00107 | 37.72424 |
| 350 | 0.00107 | 37.72424 |
| 350 | 0.00117 | 37.91595 |
| 350 | 0.00127 | 38.06801 |
| 350 | 0.00136 | 38.18039 |
| 350 | 0.0015 | 38.18039 |
| 350 | 0.00175 | 38.37211 |
| 400 | 3.30E-07 | 15.25364 |
| 400 | 4.55E-07 | 15.36602 |
| 400 | 6.37E-07 | 15.44536 |
| 400 | 8.19E-07 | 15.40569 |
| 400 | 1.23E-06 | 15.33297 |
| 400 | 1.55E-06 | 16.39733 |
| 400 | 1.80E-06 | 16.92621 |
| 400 | 2.36E-06 | 17.11792 |
| 400 | 2.99E-06 | 18.40706 |
| 400 | 3.36E-06 | 18.79049 |
| 400 | 3.66E-06 | 18.94255 |
| 400 | 4.40E-06 | 19.96724 |
| 400 | 5.38E-06 | 20.61511 |
| 400 | 5.86E-06 | 21.48776 |
| 400 | 5.86E-06 | 21.60676 |
| 400 | 6.15E-06 | 21.87119 |
| 400 | 6.36E-06 | 22.40007 |
| 400 | 6.92E-06 | 22.67112 |
| 400 | 7.17E-06 | 23.08761 |
| 400 | 7.66E-06 | 23.31238 |
| 400 | 7.17E-06 | 23.69581 |
| 400 | 7.53E-06 | 23.96025 |
| 400 | 6.58E-06 | 24.56846 |
| 400 | 7.04E-06 | 24.91223 |
| 400 | 7.53E-06 | 25.52043 |
| 400 | 8.32E-06 | 26.32036 |
| 400 | 9.05E-06 | 26.96823 |
| 400 | 1.02E-05 | 27.23267 |
| 400 | 1.09E-05 | 27.49711 |
| 400 | 1.23E-05 | 27.68882 |
| 400 | 1.33E-05 | 27.68882 |
| 400 | 1.50E-05 | 28.10531 |
| 400 | 1.58E-05 | 28.29703 |
| 400 | 1.63E-05 | 28.60113 |
| 400 | 1.69E-05 | 28.9449 |
| 400 | 1.80E-05 | 29.01762 |
| 400 | 1.87E-05 | 29.09696 |
| 400 | 1.96E-05 | 29.16968 |
| 400 | 2.21E-05 | 29.28206 |
| 400 | 2.29E-05 | 29.40106 |
| 400 | 2.45E-05 | 29.58617 |
| 400 | 2.53E-05 | 29.77788 |
| 400 | 3.04E-05 | 30.04232 |
| 400 | 3.25E-05 | 30.42576 |
| 400 | 3.97E-05 | 30.34642 |
| 400 | 4.25E-05 | 30.65053 |
| 400 | 4.55E-05 | 30.92158 |
| 400 | 4.19E-05 | 31.22568 |
| 400 | 4.95E-05 | 31.11329 |
| 400 | 6.59E-05 | 31.2984 |
| 400 | 7.67E-05 | 31.7215 |
| 400 | 9.52E-05 | 32.13799 |
| 400 | 1.04E-04 | 32.66687 |
| 400 | 1.19E-04 | 32.81892 |
| 400 | 1.33E-04 | 33.12302 |
| 400 | 1.45E-04 | 33.27507 |
| 400 | 1.55E-04 | 33.39407 |
| 400 | 1.63E-04 | 33.57918 |
| 400 | 1.77E-04 | 33.77089 |
| 400 | 1.97E-04 | 33.85022 |
| 400 | 2.07E-04 | 34.18738 |
| 400 | 2.07E-04 | 34.18738 |
| 400 | 2.17E-04 | 34.33943 |
| 400 | 2.44E-04 | 34.61048 |
| 400 | 2.66E-04 | 34.72287 |
| 400 | 2.84E-04 | 34.94764 |
| 400 | 3.04E-04 | 35.29141 |
| 400 | 3.54E-04 | 35.33108 |
| 400 | 3.85E-04 | 35.52279 |
| 400 | 4.25E-04 | 35.8269 |
| 400 | 4.41E-04 | 35.85995 |
| 400 | 4.63E-04 | 35.85995 |
| 400 | 5.49E-04 | 36.131 |
| 400 | 5.77E-04 | 36.35577 |
| 400 | 6.28E-04 | 36.35577 |
| 400 | 6.93E-04 | 36.62682 |

**Table. 1(b).** Fig.1 (b)’s data

| Temperature, ℃ | da/dN, mm/N | $\Delta$K |
| --- | --- | --- |
| 25 | 4.99E-07 | 23.56847 |
| 25 | 6.31E-07 | 23.60838 |
| 25 | 7.87E-07 | 23.45539 |
| 25 | 1.31E-06 | 23.87445 |
| 25 | 1.63E-06 | 23.98753 |
| 25 | 9.65E-07 | 24.18044 |
| 25 | 1.20E-06 | 24.4066 |
| 25 | 1.24E-06 | 24.71259 |
| 25 | 1.45E-06 | 25.0984 |
| 25 | 1.60E-06 | 25.40438 |
| 25 | 1.77E-06 | 25.47755 |
| 25 | 1.86E-06 | 25.67046 |
| 25 | 2.03E-06 | 25.75028 |
| 25 | 2.10E-06 | 25.90327 |
| 25 | 2.17E-06 | 25.97644 |
| 25 | 2.58E-06 | 26.08952 |
| 25 | 3.20E-06 | 26.39551 |
| 25 | 3.49E-06 | 26.5485 |
| 25 | 3.80E-06 | 26.82123 |
| 25 | 3.73E-06 | 27.0873 |
| 25 | 4.07E-06 | 27.0873 |
| 25 | 4.57E-06 | 27.04739 |
| 25 | 5.33E-06 | 27.12721 |
| 25 | 5.60E-06 | 27.24029 |
| 25 | 6.53E-06 | 27.58619 |
| 25 | 6.65E-06 | 27.92544 |
| 25 | 7.61E-06 | 28.31124 |
| 25 | 8.14E-06 | 28.57732 |
| 25 | 9.46E-06 | 28.65714 |
| 25 | 1.16E-05 | 28.8833 |
| 25 | 1.42E-05 | 28.8833 |
| 25 | 1.60E-05 | 28.84339 |
| 25 | 1.93E-05 | 29.11612 |
| 25 | 2.17E-05 | 29.34228 |
| 25 | 2.48E-05 | 29.60836 |
| 25 | 2.80E-05 | 29.72809 |
| 25 | 3.00E-05 | 29.84117 |
| 25 | 3.10E-05 | 30.14716 |
| 25 | 3.55E-05 | 30.34006 |
| 25 | 3.99E-05 | 30.45314 |
| 25 | 4.21E-05 | 30.52631 |
| 25 | 4.97E-05 | 30.64605 |
| 25 | 5.23E-05 | 30.79904 |
| 25 | 5.90E-05 | 30.95203 |
| 25 | 6.31E-05 | 31.13828 |
| 25 | 7.47E-05 | 31.41101 |
| 25 | 8.13E-05 | 31.717 |
| 25 | 1.05E-04 | 32.17597 |
| 25 | 1.05E-04 | 32.17597 |
| 25 | 1.24E-04 | 32.28906 |
| 25 | 1.44E-04 | 32.70812 |
| 25 | 1.71E-04 | 32.70812 |
| 25 | 2.06E-04 | 32.8212 |
| 25 | 1.99E-04 | 33.1671 |
| 25 | 2.36E-04 | 33.66599 |
| 25 | 2.10E-04 | 33.513 |
| 25 | 2.70E-04 | 33.58617 |
| 25 | 3.04E-04 | 33.89215 |
| 25 | 3.04E-04 | 34.23805 |
| 25 | 3.72E-04 | 34.47087 |
| 25 | 4.20E-04 | 34.69703 |
| 25 | 4.41E-04 | 34.85002 |
| 25 | 4.81E-04 | 35.15601 |
| 25 | 5.31E-04 | 35.309 |
| 25 | 6.42E-04 | 35.61498 |
| 25 | 6.86E-04 | 35.72807 |
| 25 | 6.74E-04 | 36.18704 |
| 25 | 6.86E-04 | 36.41986 |
| 25 | 6.63E-04 | 36.83893 |
| 25 | 6.97E-04 | 37.25799 |
| 350 | 2.25E-07 | 20.96759 |
| 350 | 6.43E-07 | 20.74143 |
| 350 | 9.18E-07 | 21.12058 |
| 350 | 1.05E-06 | 21.38666 |
| 350 | 1.42E-06 | 21.50639 |
| 350 | 1.81E-06 | 22.27135 |
| 350 | 2.44E-06 | 22.65051 |
| 350 | 2.44E-06 | 23.22257 |
| 350 | 2.90E-06 | 23.48865 |
| 350 | 3.26E-06 | 23.76137 |
| 350 | 3.43E-06 | 23.83454 |
| 350 | 4.00E-06 | 24.18044 |
| 350 | 4.28E-06 | 24.25361 |
| 350 | 5.24E-06 | 24.9454 |
| 350 | 5.51E-06 | 25.2913 |
| 350 | 6.21E-06 | 25.71037 |
| 350 | 5.81E-06 | 25.67046 |
| 350 | 6.21E-06 | 26.05626 |
| 350 | 6.75E-06 | 26.28243 |
| 350 | 6.30E-06 | 26.70149 |
| 350 | 7.61E-06 | 27.24029 |
| 350 | 7.61E-06 | 27.46646 |
| 350 | 8.14E-06 | 27.73918 |
| 350 | 8.14E-06 | 28.35115 |
| 350 | 9.00E-06 | 28.65714 |
| 350 | 9.32E-06 | 28.81013 |
| 350 | 1.05E-05 | 29.26911 |
| 350 | 1.26E-05 | 29.76135 |
| 350 | 1.24E-05 | 30.26024 |
| 350 | 1.42E-05 | 30.45314 |
| 350 | 1.68E-05 | 30.49305 |
| 350 | 1.83E-05 | 30.60613 |
| 350 | 2.10E-05 | 30.75913 |
| 350 | 2.29E-05 | 31.13828 |
| 350 | 3.10E-05 | 31.59726 |
| 350 | 3.21E-05 | 31.67708 |
| 350 | 3.48E-05 | 31.79017 |
| 350 | 3.93E-05 | 32.05624 |
| 350 | 4.13E-05 | 32.20923 |
| 350 | 4.42E-05 | 32.28906 |
| 350 | 5.23E-05 | 32.59504 |
| 350 | 5.42E-05 | 32.90103 |
| 350 | 5.70E-05 | 33.09393 |
| 350 | 6.31E-05 | 33.43318 |
| 350 | 6.52E-05 | 33.513 |
| 350 | 6.86E-05 | 34.04515 |
| 350 | 7.60E-05 | 34.16488 |
| 350 | 8.85E-05 | 34.43095 |
| 350 | 9.79E-05 | 34.58395 |
| 350 | 1.18E-04 | 34.77685 |
| 350 | 1.54E-04 | 35.00301 |
| 350 | 1.65E-04 | 34.92984 |
| 350 | 1.99E-04 | 35.15601 |
| 350 | 2.32E-04 | 35.54181 |
| 350 | 2.94E-04 | 35.57507 |
| 350 | 3.20E-04 | 35.80789 |
| 350 | 3.37E-04 | 35.88106 |
| 350 | 4.20E-04 | 36.34004 |
| 350 | 4.81E-04 | 36.37995 |
| 350 | 4.72E-04 | 36.72585 |
| 350 | 6.08E-04 | 36.91875 |
| 350 | 7.72E-04 | 37.105 |
| 350 | 8.53E-04 | 37.22473 |
| 350 | 9.13E-04 | 37.53072 |
| 350 | 0.00116 | 37.79679 |
| 350 | 0.00133 | 37.86997 |
| 350 | 0.00145 | 38.06287 |
| 400 | 3.67E-07 | 22.27135 |
| 400 | 4.75E-07 | 22.15162 |
| 400 | 8.86E-07 | 22.30461 |
| 400 | 1.42E-06 | 22.73033 |
| 400 | 1.58E-06 | 22.88333 |
| 400 | 2.17E-06 | 23.02967 |
| 400 | 2.76E-06 | 23.1494 |
| 400 | 3.26E-06 | 23.64164 |
| 400 | 3.80E-06 | 24.10062 |
| 400 | 4.66E-06 | 24.83232 |
| 400 | 5.43E-06 | 25.17157 |
| 400 | 6.42E-06 | 25.71037 |
| 400 | 6.87E-06 | 26.05626 |
| 400 | 7.86E-06 | 26.62832 |
| 400 | 8.00E-06 | 26.93431 |
| 400 | 8.14E-06 | 27.54628 |
| 400 | 9.00E-06 | 28.07843 |
| 400 | 8.14E-06 | 28.92321 |
| 400 | 9.63E-06 | 29.38219 |
| 400 | 1.05E-05 | 29.80126 |
| 400 | 1.26E-05 | 29.99416 |
| 400 | 1.60E-05 | 30.60613 |
| 400 | 1.75E-05 | 31.06511 |
| 400 | 2.14E-05 | 31.33119 |
| 400 | 2.62E-05 | 31.52409 |
| 400 | 2.89E-05 | 31.59726 |
| 400 | 3.55E-05 | 31.83008 |
| 400 | 4.13E-05 | 32.13606 |
| 400 | 4.65E-05 | 32.48196 |
| 400 | 4.73E-05 | 32.48196 |
| 400 | 5.60E-05 | 32.70812 |
| 400 | 6.75E-05 | 33.01411 |
| 400 | 9.47E-05 | 33.36 |
| 400 | 1.18E-04 | 33.81898 |
| 400 | 1.28E-04 | 34.08506 |
| 400 | 1.40E-04 | 34.08506 |
| 400 | 1.57E-04 | 34.39104 |
| 400 | 1.77E-04 | 34.69703 |
| 400 | 2.06E-04 | 34.81011 |
| 400 | 2.48E-04 | 35.1161 |
| 400 | 2.70E-04 | 35.26909 |
| 400 | 3.04E-04 | 35.76798 |
| 400 | 3.37E-04 | 35.92097 |
| 400 | 3.79E-04 | 36.41986 |
| 400 | 3.92E-04 | 36.41986 |
| 400 | 3.92E-04 | 36.41986 |
| 400 | 4.81E-04 | 36.49303 |
| 400 | 5.05E-04 | 36.57285 |
| 400 | 6.08E-04 | 36.83893 |
| 400 | 6.30E-04 | 36.91875 |
| 400 | 6.86E-04 | 37.03183 |

**Table. 1(c).** Fig.1 (c)’s data

| Temperature, ℃ | da/dN, mm/N | $\Delta$K |
| --- | --- | --- |
| 25 | 5.15E-07 | 24.31303 |
| 25 | 6.00E-07 | 24.40588 |
| 25 | 7.56E-07 | 24.4523 |
| 25 | 9.26E-07 | 24.22018 |
| 25 | 1.11E-06 | 24.31303 |
| 25 | 1.27E-06 | 24.4523 |
| 25 | 1.39E-06 | 24.54515 |
| 25 | 1.32E-06 | 24.86348 |
| 25 | 1.18E-06 | 24.9298 |
| 25 | 1.03E-06 | 24.97623 |
| 25 | 1.16E-06 | 25.32109 |
| 25 | 1.21E-06 | 25.48026 |
| 25 | 1.22E-06 | 25.66596 |
| 25 | 1.30E-06 | 25.82513 |
| 25 | 1.46E-06 | 26.14346 |
| 25 | 1.68E-06 | 26.39548 |
| 25 | 1.78E-06 | 26.53475 |
| 25 | 1.95E-06 | 26.71382 |
| 25 | 2.47E-06 | 27.17142 |
| 25 | 2.84E-06 | 27.2908 |
| 25 | 3.21E-06 | 27.37702 |
| 25 | 3.51E-06 | 27.62903 |
| 25 | 4.14E-06 | 27.81473 |
| 25 | 4.48E-06 | 28.02032 |
| 25 | 5.11E-06 | 28.27234 |
| 25 | 5.71E-06 | 28.49783 |
| 25 | 6.07E-06 | 28.68352 |
| 25 | 6.79E-06 | 28.93554 |
| 25 | 7.88E-06 | 29.18756 |
| 25 | 9.96E-06 | 29.55232 |
| 25 | 1.11E-05 | 29.82423 |
| 25 | 1.32E-05 | 29.87065 |
| 25 | 1.67E-05 | 30.44764 |
| 25 | 2.06E-05 | 30.58028 |
| 25 | 2.63E-05 | 31.13073 |
| 25 | 2.88E-05 | 31.2899 |
| 25 | 3.31E-05 | 31.4756 |
| 25 | 3.94E-05 | 31.70109 |
| 25 | 4.49E-05 | 31.97963 |
| 25 | 5.17E-05 | 32.1388 |
| 25 | 6.14E-05 | 32.34439 |
| 25 | 7.37E-05 | 32.54998 |
| 25 | 8.24E-05 | 32.77547 |
| 25 | 9.67E-05 | 33.00759 |
| 25 | 1.08E-04 | 33.14687 |
| 25 | 1.17E-04 | 33.30603 |
| 25 | 1.34E-04 | 33.53152 |
| 25 | 1.51E-04 | 33.85649 |
| 25 | 1.70E-04 | 34.08198 |
| 25 | 2.00E-04 | 34.40032 |
| 25 | 2.17E-04 | 34.65233 |
| 25 | 2.41E-04 | 34.9773 |
| 25 | 2.83E-04 | 35.29564 |
| 25 | 3.03E-04 | 35.61397 |
| 25 | 3.42E-04 | 35.95884 |
| 25 | 3.79E-04 | 36.05169 |
| 25 | 4.54E-04 | 36.37002 |
| 25 | 5.02E-04 | 36.64193 |
| 25 | 5.45E-04 | 36.76131 |
| 25 | 6.09E-04 | 37.07965 |
| 25 | 6.14E-04 | 37.37809 |
| 25 | 6.27E-04 | 37.78927 |
| 350 | 2.18E-07 | 15.66489 |
| 350 | 6.31E-07 | 15.53225 |
| 350 | 8.21E-07 | 15.48582 |
| 350 | 1.03E-06 | 15.55214 |
| 350 | 1.13E-06 | 16.07607 |
| 350 | 1.42E-06 | 16.32809 |
| 350 | 1.56E-06 | 17.2433 |
| 350 | 1.91E-06 | 17.79376 |
| 350 | 2.23E-06 | 18.64266 |
| 350 | 2.84E-06 | 19.30586 |
| 350 | 3.58E-06 | 20.08181 |
| 350 | 3.78E-06 | 20.77153 |
| 350 | 4.17E-06 | 21.13629 |
| 350 | 4.62E-06 | 21.97856 |
| 350 | 5.00E-06 | 22.55555 |
| 350 | 5.78E-06 | 23.1259 |
| 350 | 6.13E-06 | 23.76257 |
| 350 | 6.85E-06 | 24.15386 |
| 350 | 7.28E-06 | 24.59157 |
| 350 | 8.31E-06 | 25.00276 |
| 350 | 8.91E-06 | 25.7986 |
| 350 | 9.10E-06 | 26.23631 |
| 350 | 1.04E-05 | 26.74034 |
| 350 | 1.19E-05 | 27.03878 |
| 350 | 1.35E-05 | 27.2908 |
| 350 | 1.48E-05 | 27.58261 |
| 350 | 1.72E-05 | 27.83463 |
| 350 | 1.90E-05 | 28.08664 |
| 350 | 2.21E-05 | 28.29223 |
| 350 | 2.55E-05 | 28.74984 |
| 350 | 3.06E-05 | 29.25388 |
| 350 | 3.09E-05 | 29.69159 |
| 350 | 3.28E-05 | 30.00992 |
| 350 | 3.56E-05 | 30.37468 |
| 350 | 4.05E-05 | 30.76597 |
| 350 | 4.72E-05 | 31.08431 |
| 350 | 5.55E-05 | 31.38275 |
| 350 | 6.20E-05 | 31.74751 |
| 350 | 7.01E-05 | 32.02606 |
| 350 | 8.31E-05 | 32.45714 |
| 350 | 9.96E-05 | 32.77547 |
| 350 | 1.16E-04 | 33.02749 |
| 350 | 1.44E-04 | 33.35246 |
| 350 | 1.76E-04 | 33.69069 |
| 350 | 2.04E-04 | 34.03555 |
| 350 | 2.55E-04 | 34.33399 |
| 350 | 2.79E-04 | 34.63244 |
| 350 | 3.12E-04 | 34.8115 |
| 350 | 3.71E-04 | 35.18289 |
| 350 | 4.15E-04 | 35.40838 |
| 350 | 4.98E-04 | 35.72672 |
| 350 | 5.79E-04 | 35.93231 |
| 350 | 6.95E-04 | 36.16443 |
| 350 | 8.41E-04 | 36.38992 |
| 350 | 9.99E-04 | 36.50929 |
| 350 | 0.00113 | 36.66846 |
| 350 | 0.00127 | 36.94037 |
| 350 | 0.00161 | 37.05312 |
| 400 | 3.58E-07 | 17.33615 |
| 400 | 5.11E-07 | 16.7658 |
| 400 | 7.65E-07 | 17.08414 |
| 400 | 1.03E-06 | 17.19688 |
| 400 | 1.48E-06 | 17.52185 |
| 400 | 1.62E-06 | 18.13863 |
| 400 | 2.21E-06 | 18.2779 |
| 400 | 2.87E-06 | 18.91457 |
| 400 | 3.41E-06 | 19.21301 |
| 400 | 4.01E-06 | 19.78336 |
| 400 | 4.26E-06 | 20.22108 |
| 400 | 4.76E-06 | 20.67869 |
| 400 | 5.59E-06 | 21.38831 |
| 400 | 6.25E-06 | 22.00509 |
| 400 | 6.13E-06 | 22.78103 |
| 400 | 6.79E-06 | 23.58351 |
| 400 | 7.50E-06 | 24.24671 |
| 400 | 7.59E-06 | 24.54515 |
| 400 | 7.20E-06 | 24.77064 |
| 400 | 6.71E-06 | 25.20835 |
| 400 | 6.79E-06 | 25.63943 |
| 400 | 7.74E-06 | 26.00419 |
| 400 | 7.97E-06 | 26.58118 |
| 400 | 8.91E-06 | 26.96583 |
| 400 | 1.01E-05 | 27.19795 |
| 400 | 1.12E-05 | 27.26427 |
| 400 | 1.27E-05 | 27.56271 |
| 400 | 1.42E-05 | 27.62903 |
| 400 | 1.59E-05 | 27.92747 |
| 400 | 1.70E-05 | 28.27234 |
| 400 | 1.90E-05 | 28.57078 |
| 400 | 2.08E-05 | 28.84269 |
| 400 | 2.28E-05 | 29.07481 |
| 400 | 2.68E-05 | 29.34672 |
| 400 | 3.12E-05 | 29.50589 |
| 400 | 4.44E-05 | 30.07624 |
| 400 | 5.12E-05 | 30.44764 |
| 400 | 6.40E-05 | 30.92514 |
| 400 | 7.22E-05 | 31.13073 |
| 400 | 8.58E-05 | 31.52202 |
| 400 | 9.88E-05 | 31.84036 |
| 400 | 1.09E-04 | 32.09238 |
| 400 | 1.32E-04 | 32.39082 |
| 400 | 1.57E-04 | 32.56988 |
| 400 | 1.67E-04 | 33.07391 |
| 400 | 1.83E-04 | 33.19329 |
| 400 | 2.13E-04 | 33.51163 |
| 400 | 2.48E-04 | 33.71722 |
| 400 | 2.41E-04 | 34.22125 |
| 400 | 2.66E-04 | 34.53959 |
| 400 | 3.23E-04 | 34.9773 |
| 400 | 3.79E-04 | 35.18289 |
| 400 | 4.19E-04 | 35.22932 |
| 400 | 5.02E-04 | 35.43491 |
| 400 | 6.27E-04 | 35.54765 |

**Fig. 2.** The relationship between stress and cyclic cycles under different strain amplitudes.

**Table. 2.** Fig.2’s data

| Strain amplitude, % | Stress amplitude, MPa | Fatigue life, N |
| --- | --- | --- |
| 0.2 | 230.86206 | 1.03391 |
| 0.2 | 233.11098 | 1.12615 |
| 0.2 | 234.34426 | 1.21563 |
| 0.2 | 237.101 | 1.33605 |
| 0.2 | 238.11664 | 1.46509 |
| 0.2 | 240.65574 | 1.60659 |
| 0.2 | 241.59884 | 1.74992 |
| 0.2 | 243.1223 | 1.93628 |
| 0.2 | 243.84776 | 2.12808 |
| 0.2 | 243.94756 | 2.38669 |
| 0.2 | 244.35558 | 2.68276 |
| 0.2 | 244.36258 | 2.87646 |
| 0.2 | 244.64576 | 3.21155 |
| 0.2 | 244.43576 | 3.52173 |
| 0.2 | 244.35558 | 4.02141 |
| 0.2 | 244.34554 | 4.43968 |
| 0.2 | 244.34358 | 5.26716 |
| 0.2 | 244.32108 | 5.88075 |
| 0.2 | 244.31258 | 6.70005 |
| 0.2 | 244.30518 | 7.70247 |
| 0.2 | 244.32558 | 8.7362 |
| 0.2 | 241.59884 | 9.79787 |
| 0.2 | 242.39684 | 11.11281 |
| 0.2 | 241.59884 | 12.66104 |
| 0.2 | 242.39684 | 13.79056 |
| 0.2 | 242.39684 | 15.15658 |
| 0.2 | 242.61448 | 16.733 |
| 0.2 | 242.71448 | 18.39048 |
| 0.2 | 242.90466 | 20.81175 |
| 0.2 | 242.93266 | 23.18397 |
| 0.2 | 242.10666 | 25.88472 |
| 0.2 | 242.10126 | 28.83517 |
| 0.2 | 241.59884 | 31.62024 |
| 0.2 | 241.16356 | 35.54274 |
| 0.2 | 240.87338 | 39.32783 |
| 0.2 | 240.65574 | 44.90785 |
| 0.2 | 239.6401 | 50.02665 |
| 0.2 | 238.62446 | 59.35081 |
| 0.2 | 238.40682 | 67.16489 |
| 0.2 | 238.30543 | 89.56808 |
| 0.2 | 235.3599 | 101.36053 |
| 0.2 | 235.65008 | 113.93433 |
| 0.2 | 235.3599 | 141.38809 |
| 0.2 | 235.65008 | 157.50412 |
| 0.2 | 234.34426 | 165.12071 |
| 0.2 | 234.12662 | 203.53065 |
| 0.2 | 233.40116 | 218.2257 |
| 0.2 | 232.89334 | 243.64733 |
| 0.2 | 233.11098 | 334.55617 |
| 0.2 | 231.8777 | 372.69036 |
| 0.2 | 232.38552 | 412.37974 |
| 0.2 | 228.61314 | 677.85462 |
| 0.2 | 225.34858 | 791.63554 |
| 0.2 | 227.88768 | 835.53536 |
| 0.2 | 228.61314 | 924.51508 |
| 0.2 | 227.88768 | 1269.46692 |
| 0.2 | 227.5975 | 1426.94453 |
| 0.2 | 226.58186 | 1554.24586 |
| 0.2 | 225.8564 | 1747.05035 |
| 0.2 | 225.63877 | 1902.90913 |
| 0.2 | 225.13095 | 2202.42019 |
| 0.2 | 224.84076 | 2498.00023 |
| 0.2 | 224.1153 | 2720.85315 |
| 0.2 | 223.82512 | 2963.58735 |
| 0.2 | 222.88203 | 3199.07019 |
| 0.2 | 223.60748 | 3881.64226 |
| 0.2 | 223.82512 | 4432.38776 |
| 0.2 | 222.08402 | 6525.61165 |
| 0.2 | 221.86639 | 9041.39001 |
| 0.2 | 221.5762 | 9694.18482 |
| 0.2 | 220.12529 | 10324.22458 |
| 0.2 | 220.34292 | 12640.25323 |
| 0.2 | 220.85074 | 14304.45848 |
| 0.2 | 222.59184 | 17513.37121 |
| 0.2 | 219.8351 | 19509.62237 |
| 0.2 | 219.10965 | 28982.70616 |
| 0.2 | 216.57054 | 46164.17687 |
| 0.2 | 216.13527 | 52242.11409 |
| 0.2 | 215.62745 | 57805.59943 |
| 0.2 | 213.8138 | 70139.33566 |
| 0.2 | 208.59051 | 77086.95117 |
| 0.2 | 194.29899 | 75882.97211 |
| 0.3 | 261.40383 | 1.08391 |
| 0.3 | 262.92729 | 1.21563 |
| 0.3 | 265.39385 | 1.39751 |
| 0.3 | 267.93295 | 1.56031 |
| 0.3 | 268.15059 | 1.71101 |
| 0.3 | 267.93295 | 2.14248 |
| 0.3 | 270.68969 | 2.26129 |
| 0.3 | 272.93861 | 2.55901 |
| 0.3 | 272.43079 | 2.90245 |
| 0.3 | 274.38953 | 3.01556 |
| 0.3 | 275.40517 | 3.28458 |
| 0.3 | 274.17189 | 3.71703 |
| 0.3 | 276.42081 | 4.11287 |
| 0.3 | 276.92863 | 4.65437 |
| 0.3 | 277.65409 | 5.31475 |
| 0.3 | 280.19319 | 5.97405 |
| 0.3 | 280.41083 | 6.86785 |
| 0.3 | 280.31083 | 7.89538 |
| 0.3 | 280.19319 | 8.79533 |
| 0.3 | 280.70101 | 10.50528 |
| 0.3 | 279.39519 | 13.06599 |
| 0.3 | 281.42647 | 16.47165 |
| 0.3 | 278.88737 | 19.85176 |
| 0.3 | 278.66973 | 24.3051 |
| 0.3 | 276.20317 | 29.75745 |
| 0.3 | 275.40517 | 37.26151 |
| 0.3 | 273.15625 | 44.5057 |
| 0.3 | 270.18187 | 54.48965 |
| 0.3 | 269.38387 | 62.64208 |
| 0.3 | 266.40949 | 74.31756 |
| 0.3 | 267.93295 | 84.67146 |
| 0.3 | 266.19185 | 99.77744 |
| 0.3 | 263.65275 | 114.70558 |
| 0.3 | 263.65275 | 142.66565 |
| 0.3 | 261.62147 | 157.50412 |
| 0.3 | 260.38819 | 181.47665 |
| 0.3 | 259.15491 | 213.37298 |
| 0.3 | 257.63145 | 263.00724 |
| 0.3 | 257.12363 | 314.13975 |
| 0.3 | 255.67272 | 347.59384 |
| 0.3 | 255.1649 | 409.60701 |
| 0.3 | 254.14926 | 500.36746 |
| 0.3 | 252.11797 | 562.43814 |
| 0.3 | 251.17488 | 656.84589 |
| 0.3 | 249.65142 | 726.79621 |
| 0.3 | 249.45142 | 809.63964 |
| 0.3 | 249.1436 | 945.54126 |
| 0.3 | 247.4025 | 1131.91114 |
| 0.3 | 247.11232 | 1321.90746 |
| 0.3 | 247.10232 | 1519.68381 |
| 0.3 | 246.38686 | 1692.90407 |
| 0.3 | 246.16922 | 1802.92847 |
| 0.3 | 244.8634 | 2072.67247 |
| 0.3 | 244.13794 | 2237.36436 |
| 0.3 | 243.1223 | 2498.00023 |
| 0.3 | 242.39684 | 2757.81413 |
| 0.3 | 241.3812 | 3051.5055 |
| 0.3 | 240.65574 | 3539.7528 |
| 0.3 | 240.65574 | 4096.89716 |
| 0.3 | 240.36556 | 4363.16071 |
| 0.3 | 240.32556 | 4784.57959 |
| 0.3 | 239.85774 | 5258.51364 |
| 0.3 | 239.34992 | 5766.41079 |
| 0.3 | 237.39118 | 6902.99292 |
| 0.3 | 236.59318 | 7689.82645 |
| 0.3 | 236.1579 | 8643.75087 |
| 0.3 | 236.1239 | 10004.24616 |
| 0.3 | 234.85208 | 12248.4942 |
| 0.3 | 233.11098 | 13552.88854 |
| 0.4 | 272.14061 | 1.09863 |
| 0.4 | 273.44643 | 1.33605 |
| 0.4 | 275.69535 | 1.52218 |
| 0.4 | 276.20317 | 1.71101 |
| 0.4 | 277.14627 | 1.95378 |
| 0.4 | 279.68537 | 2.33362 |
| 0.4 | 279.39519 | 2.48528 |
| 0.4 | 280.70101 | 2.83153 |
| 0.4 | 282.15193 | 3.08414 |
| 0.4 | 283.45775 | 3.44342 |
| 0.4 | 284.90866 | 3.80157 |
| 0.4 | 285.92431 | 4.17813 |
| 0.4 | 287.15758 | 4.72822 |
| 0.4 | 288.46341 | 5.31475 |
| 0.4 | 289.69669 | 5.82808 |
| 0.4 | 290.20451 | 6.49239 |
| 0.4 | 290.20451 | 7.36372 |
| 0.4 | 291.65542 | 8.2586 |
| 0.4 | 292.96125 | 9.28308 |
| 0.4 | 294.19452 | 10.93924 |
| 0.4 | 294.91998 | 12.46329 |
| 0.4 | 296.22581 | 13.79056 |
| 0.4 | 295.93562 | 15.74723 |
| 0.4 | 295.71799 | 17.70068 |
| 0.4 | 295.21017 | 19.41031 |
| 0.4 | 292.45343 | 23.39345 |
| 0.4 | 290.92996 | 27.50508 |
| 0.4 | 290.71233 | 31.62024 |
| 0.4 | 289.69669 | 37.51374 |
| 0.4 | 289.18887 | 45.51789 |
| 0.4 | 287.6654 | 52.44583 |
| 0.4 | 286.43213 | 56.61311 |
| 0.4 | 285.70667 | 67.61954 |
| 0.4 | 283.67539 | 76.69455 |
| 0.4 | 281.6441 | 89.56808 |
| 0.4 | 280.19319 | 102.96875 |
| 0.4 | 278.45209 | 117.31432 |
| 0.4 | 277.65409 | 127.78023 |
| 0.4 | 277.55409 | 138.24401 |
| 0.4 | 277.25402 | 154.00167 |
| 0.4 | 274.89735 | 182.70511 |
| 0.4 | 274.38953 | 206.75992 |
| 0.4 | 272.64843 | 228.77866 |
| 0.4 | 270.90733 | 281.99655 |
| 0.4 | 269.67405 | 334.55617 |
| 0.4 | 268.44077 | 394.24334 |
| 0.4 | 268.15059 | 425.5694 |
| 0.4 | 267.42513 | 500.36746 |
| 0.4 | 265.90167 | 553.65373 |
| 0.4 | 264.88603 | 627.95789 |
| 0.4 | 262.92729 | 822.48559 |
| 0.4 | 261.40383 | 895.86161 |
| 0.4 | 259.88037 | 984.60072 |
| 0.4 | 260.17056 | 1149.87034 |
| 0.4 | 257.63145 | 1289.60861 |
| 0.4 | 257.61145 | 1449.5848 |
| 0.4 | 256.18054 | 1731.40565 |
| 0.4 | 256.12054 | 1885.86873 |
| 0.4 | 254.87471 | 2172.90275 |
| 0.4 | 255.1649 | 2636.52582 |
| 0.4 | 251.39252 | 3149.10565 |
| 0.4 | 250.66706 | 4096.89716 |
| 0.4 | 249.1436 | 4752.40932 |
| 0.4 | 246.89468 | 5550.12238 |
| 0.4 | 244.13794 | 7107.77798 |
| 0.4 | 241.16356 | 8900.17747 |
| 0.4 | 235.65008 | 8840.33499 |
| 0.4 | 229.62878 | 8432.55362 |
| 0.4 | 222.37421 | 8900.17747 |
| 0.4 | 208.37287 | 8432.55362 |
| 0.5 | 304.71366 | 1.0742 |
| 0.5 | 305.43912 | 1.28304 |
| 0.5 | 305.7293 | 1.44221 |
| 0.5 | 305.7293 | 1.73816 |
| 0.5 | 308.70368 | 1.99821 |
| 0.5 | 310.73496 | 2.31793 |
| 0.5 | 313.92698 | 2.66472 |
| 0.5 | 313.92698 | 2.9221 |
| 0.5 | 316.68371 | 3.13308 |
| 0.5 | 320.74628 | 3.65898 |
| 0.5 | 321.18155 | 4.47979 |
| 0.5 | 322.19719 | 5.11541 |
| 0.5 | 323.50302 | 5.60948 |
| 0.5 | 323.93829 | 6.70005 |
| 0.5 | 325.24412 | 7.58217 |
| 0.5 | 326.98522 | 8.38963 |
| 0.5 | 328.00086 | 9.51558 |
| 0.5 | 328.00086 | 10.76839 |
| 0.5 | 328.00086 | 12.07702 |
| 0.5 | 328.00086 | 13.57518 |
| 0.5 | 328.21849 | 15.50129 |
| 0.5 | 328.21849 | 17.42422 |
| 0.5 | 327.49304 | 19.85176 |
| 0.5 | 327.71067 | 22.31437 |
| 0.5 | 328.50868 | 24.85786 |
| 0.5 | 329.74195 | 28.38481 |
| 0.5 | 328.00086 | 31.90596 |
| 0.5 | 325.96957 | 36.92784 |
| 0.5 | 324.95393 | 41.50874 |
| 0.5 | 324.22848 | 46.24009 |
| 0.5 | 323.72066 | 51.62671 |
| 0.5 | 322.9952 | 57.51135 |
| 0.5 | 321.68937 | 62.64208 |
| 0.5 | 319.44045 | 70.41285 |
| 0.5 | 317.19154 | 78.96978 |
| 0.5 | 315.2328 | 93.05855 |
| 0.5 | 315.2328 | 108.67884 |
| 0.5 | 315.45044 | 122.9874 |
| 0.5 | 315.95826 | 136.08486 |
| 0.5 | 315.74062 | 158.9273 |
| 0.5 | 311.67806 | 181.47665 |
| 0.5 | 312.18588 | 232.40852 |
| 0.5 | 309.2115 | 243.64733 |
| 0.5 | 306.45476 | 281.99655 |
| 0.5 | 306.45476 | 339.86433 |
| 0.5 | 302.68238 | 460.41884 |
| 0.5 | 303.48038 | 529.30411 |
| 0.5 | 302.68238 | 656.84589 |
| 0.5 | 300.94128 | 767.1004 |
| 0.5 | 299.20018 | 903.95648 |
| 0.5 | 297.9669 | 1029.89538 |
| 0.5 | 294.41216 | 1280.93762 |
| 0.5 | 288.89868 | 1373.42223 |
| 0.5 | 281.20883 | 1449.5848 |
| 0.5 | 273.88171 | 1506.07516 |
| 0.5 | 268.87605 | 1543.79552 |
| 0.5 | 261.91165 | 1629.40607 |
| 0.5 | 257.41382 | 1529.97094 |
| 0.5 | 248.85341 | 1593.17267 |
| 0.5 | 244.13794 | 1593.17267 |
| 0.5 | 236.88336 | 1578.90593 |
| 0.5 | 228.39551 | 1554.24586 |
| 0.5 | 227.37986 | 1873.18863 |
| 0.6 | 283.16757 | 1.09863 |
| 0.6 | 285.41649 | 1.20746 |
| 0.6 | 287.6654 | 1.3034 |
| 0.6 | 290.71233 | 1.43251 |
| 0.6 | 294.19452 | 1.57087 |
| 0.6 | 298.18454 | 1.86365 |
| 0.6 | 299.41782 | 2.07608 |
| 0.6 | 301.4491 | 2.28173 |
| 0.6 | 301.95692 | 2.46303 |
| 0.6 | 303.91566 | 2.64086 |
| 0.6 | 305.7293 | 3.11201 |
| 0.6 | 306.6724 | 3.35929 |
| 0.6 | 308.92132 | 3.75062 |
| 0.6 | 311.17024 | 4.07604 |
| 0.6 | 312.47606 | 4.43968 |
| 0.6 | 314.21716 | 4.72822 |
| 0.6 | 316.68371 | 5.1849 |
| 0.6 | 317.98954 | 5.88075 |
| 0.6 | 321.18155 | 6.44874 |
| 0.6 | 321.97956 | 7.13549 |
| 0.6 | 323.21283 | 7.65068 |
| 0.6 | 323.93829 | 8.20307 |
| 0.6 | 325.75194 | 9.22066 |
| 0.6 | 326.98522 | 10.3412 |
| 0.6 | 328.00086 | 11.72906 |
| 0.6 | 328.94395 | 13.06599 |
| 0.6 | 328.97695 | 14.91986 |
| 0.6 | 330.24978 | 17.15208 |
| 0.6 | 331.19287 | 19.71828 |
| 0.6 | 331.29287 | 21.4774 |
| 0.6 | 331.34287 | 23.92549 |
| 0.6 | 331.40282 | 26.65262 |
| 0.6 | 331.79287 | 30.22959 |
| 0.6 | 327.20285 | 38.10895 |
| 0.6 | 325.96957 | 43.81059 |
| 0.6 | 324.7363 | 55.3542 |
| 0.6 | 323.93829 | 62.08113 |
| 0.6 | 323.21283 | 70.88949 |
| 0.6 | 320.23846 | 78.43881 |
| 0.6 | 319.73064 | 90.7848 |
| 0.6 | 317.69936 | 98.88394 |
| 0.6 | 316.68371 | 107.70563 |
| 0.6 | 315.95826 | 113.93433 |
| 0.6 | 315.65826 | 122.16047 |
| 0.6 | 315.45044 | 133.95942 |
| 0.6 | 314.4348 | 143.63139 |
| 0.6 | 313.20152 | 162.54178 |
| 0.6 | 312.6937 | 178.64227 |
| 0.6 | 312.18588 | 200.35181 |
| 0.6 | 311.17024 | 218.2257 |
| 0.6 | 309.93696 | 265.38373 |
| 0.6 | 310.22714 | 316.97827 |
| 0.6 | 309.2115 | 361.95263 |
| 0.6 | 309.71932 | 439.18093 |
| 0.6 | 310.44478 | 456.29582 |
| 0.6 | 310.73496 | 497.00312 |
| 0.6 | 310.53496 | 567.52024 |
| 0.6 | 310.43443 | 774.03179 |
| 0.6 | 309.93696 | 881.86964 |
| 0.6 | 308.92132 | 1079.69906 |
| 0.6 | 308.54132 | 1221.85134 |
| 0.6 | 308.48604 | 1321.90746 |
| 0.6 | 306.96258 | 1472.58429 |
| 0.6 | 306.45476 | 1692.90407 |
| 0.6 | 305.94694 | 2040.30053 |
| 0.6 | 304.20584 | 2420.57978 |
| 0.6 | 298.91 | 3003.8457 |
| 0.6 | 266.19185 | 3271.82638 |
| 0.7 | 292.45343 | 1.03391 |
| 0.7 | 292.55343 | 1.16217 |
| 0.7 | 292.67106 | 1.26585 |
| 0.7 | 292.16324 | 1.39751 |
| 0.7 | 293.46907 | 1.63209 |
| 0.7 | 292.67106 | 1.76574 |
| 0.7 | 292.97106 | 2.02991 |
| 0.7 | 293.46907 | 2.19121 |
| 0.7 | 292.16324 | 2.48528 |
| 0.7 | 293.6867 | 2.98856 |
| 0.7 | 294.70234 | 3.54557 |
| 0.7 | 294.90234 | 4.11287 |
| 0.7 | 295.21017 | 4.91248 |
| 0.7 | 296.66108 | 5.64745 |
| 0.7 | 296.76108 | 6.24888 |
| 0.7 | 296.95126 | 6.86785 |
| 0.7 | 296.95126 | 7.58217 |
| 0.7 | 297.45908 | 8.52274 |
| 0.7 | 297.55908 | 9.57999 |
| 0.7 | 297.67672 | 10.04326 |
| 0.7 | 297.67672 | 11.36554 |
| 0.7 | 298.18454 | 12.37949 |
| 0.7 | 298.91 | 13.4839 |
| 0.7 | 298.9543 | 14.55531 |
| 0.7 | 299.20018 | 17.15208 |
| 0.7 | 300.94128 | 21.62278 |
| 0.7 | 300.43346 | 24.85786 |
| 0.7 | 301.15892 | 26.89345 |
| 0.7 | 301.66674 | 29.49097 |
| 0.7 | 301.15892 | 31.62024 |
| 0.7 | 300.94128 | 34.98761 |
| 0.7 | 301.66674 | 38.10895 |
| 0.7 | 300.94128 | 46.97375 |
| 0.7 | 300.94128 | 51.97618 |
| 0.7 | 301.15892 | 57.51135 |
| 0.7 | 300.21582 | 64.64564 |
| 0.7 | 299.708 | 74.31756 |
| 0.7 | 299.41782 | 86.79209 |
| 0.7 | 296.95126 | 102.04667 |
| 0.7 | 295.21017 | 120.25251 |
| 0.7 | 295.21017 | 129.80762 |
| 0.7 | 294.91998 | 146.89798 |
| 0.7 | 293.6867 | 170.40197 |
| 0.7 | 293.46907 | 191.54037 |
| 0.7 | 293.17888 | 211.93832 |
| 0.7 | 291.94561 | 228.77866 |
| 0.7 | 291.43778 | 250.87541 |
| 0.7 | 291.33778 | 277.5922 |
| 0.7 | 291.22015 | 309.92957 |
| 0.7 | 289.91432 | 332.3067 |
| 0.7 | 288.68105 | 361.95263 |
| 0.7 | 288.68105 | 418.92267 |
| 0.7 | 288.17322 | 460.41884 |
| 0.7 | 287.44777 | 516.37135 |
| 0.7 | 286.93995 | 576.52467 |
| 0.7 | 286.43213 | 627.95789 |
| 0.7 | 286.43213 | 704.27063 |
| 0.7 | 286.21449 | 755.11947 |
| 0.7 | 285.19885 | 843.08513 |
| 0.7 | 285.41649 | 910.07559 |
| 0.7 | 285.31649 | 1029.89538 |
| 0.7 | 285.19885 | 1168.11448 |
| 0.7 | 284.90866 | 1280.93762 |
| 0.7 | 284.80865 | 1395.21331 |
| 0.7 | 284.50866 | 1568.28978 |
| 0.7 | 284.90866 | 1758.87658 |
| 0.7 | 284.69103 | 2172.90275 |
| 0.7 | 283.89302 | 2654.37314 |
| 0.7 | 283.67539 | 2963.58735 |
| 0.7 | 283.65439 | 3220.72551 |
| 0.7 | 283.57542 | 3461.03868 |
| 0.7 | 283.45775 | 3710.92814 |
| 0.7 | 282.44211 | 4005.79372 |
| 0.7 | 282.15193 | 4199.50586 |
| 0.7 | 280.19319 | 4860.49298 |
| 0.7 | 279.17755 | 5550.12238 |
| 0.7 | 279.47755 | 6182.74977 |
| 0.7 | 280.19319 | 7172.00273 |
| 0.7 | 281.93429 | 9478.61349 |
| 0.7 | 281.6441 | 11868.87694 |
| 0.7 | 281.93429 | 13552.88854 |
| 0.7 | 279.68537 | 15097.70644 |
| 0.7 | 276.92863 | 18484.56813 |
| 0.7 | 276.42081 | 20730.90983 |
| 0.7 | 273.88171 | 28084.44591 |
| 0.7 | 265.68403 | 30384.25166 |
| 0.7 | 255.89035 | 34384.61702 |
| 0.7 | 240.87338 | 32798.54521 |
| 0.7 | 192.05007 | 33544.47986 |
| 0.8 | 305.43912 | 1.03391 |
| 0.8 | 306.43912 | 1.10856 |
| 0.8 | 309.71932 | 1.3034 |
| 0.8 | 314.72498 | 1.67296 |
| 0.8 | 316.9739 | 1.89322 |
| 0.8 | 319.94827 | 2.09484 |
| 0.8 | 321.68937 | 2.22598 |
| 0.8 | 323.72066 | 2.44647 |
| 0.8 | 326.18721 | 2.59962 |
| 0.8 | 326.69503 | 2.78731 |
| 0.8 | 328.50868 | 2.9485 |
| 0.8 | 329.74195 | 3.28458 |
| 0.8 | 333.00651 | 3.52173 |
| 0.8 | 334.45743 | 3.83592 |
| 0.8 | 337.50435 | 4.38017 |
| 0.8 | 339.24545 | 4.76022 |
| 0.8 | 341.71201 | 5.0696 |
| 0.8 | 343.74329 | 5.31475 |
| 0.8 | 343.01783 | 6.15128 |
| 0.8 | 344.75893 | 6.655 |
| 0.8 | 347.73331 | 7.24871 |
| 0.8 | 349.25677 | 7.70247 |
| 0.8 | 351.50569 | 8.46544 |
| 0.8 | 353.75461 | 9.36696 |
| 0.8 | 353.97224 | 9.95332 |
| 0.8 | 355.78589 | 10.67196 |
| 0.8 | 355.49571 | 11.80846 |
| 0.8 | 356.72898 | 15.99708 |
| 0.8 | 356.51135 | 19.71828 |
| 0.8 | 355.78589 | 21.62278 |
| 0.8 | 354.77025 | 22.82187 |
| 0.8 | 353.75461 | 24.46962 |
| 0.8 | 353.75461 | 27.32015 |
| 0.8 | 353.65461 | 29.49097 |
| 0.8 | 353.53697 | 32.63159 |
| 0.8 | 352.23115 | 34.20959 |
| 0.8 | 351.50569 | 39.59405 |
| 0.8 | 349.76459 | 44.90785 |
| 0.8 | 348.96659 | 50.82038 |
| 0.8 | 347.73331 | 59.75257 |
| 0.8 | 343.23547 | 77.7364 |
| 0.8 | 343.96093 | 106.98144 |
| 0.8 | 342.51001 | 129.80762 |
| 0.8 | 342.00219 | 152.96621 |
| 0.8 | 341.49437 | 173.10561 |
| 0.8 | 343.74329 | 197.22263 |
| 0.8 | 343.23547 | 228.77866 |
| 0.8 | 342.00219 | 254.85586 |
| 0.8 | 342.10219 | 281.99655 |
| 0.8 | 342.21219 | 314.13975 |
| 0.8 | 342.24243 | 347.59384 |
| 0.8 | 342.45649 | 396.91207 |
| 0.8 | 343.01783 | 497.00312 |
| 0.8 | 343.01783 | 562.43814 |
| 0.8 | 343.23547 | 642.23949 |
| 0.8 | 343.13547 | 715.44477 |
| 0.8 | 342.51001 | 816.95541 |
| 0.8 | 340.98655 | 1013.81001 |
| 0.8 | 340.47873 | 1232.89179 |
| 0.8 | 325.46175 | 1404.65786 |
| 0.8 | 306.6724 | 1417.35012 |
| 1 | 307.4704 | 1.0742 |
| 1 | 307.6704 | 1.13377 |
| 1 | 308.48604 | 1.24608 |
| 1 | 308.70368 | 1.37568 |
| 1 | 309.71932 | 1.48833 |
| 1 | 310.73496 | 1.69951 |
| 1 | 312.18588 | 1.83455 |
| 1 | 313.70934 | 2.06212 |
| 1 | 313.90934 | 2.26129 |
| 1 | 314.94262 | 2.54181 |
| 1 | 315.2328 | 2.74995 |
| 1 | 315.95826 | 2.98856 |
| 1 | 316.68371 | 3.28458 |
| 1 | 317.98954 | 3.65898 |
| 1 | 318.49736 | 3.95861 |
| 1 | 318.93263 | 4.31176 |
| 1 | 319.94827 | 4.69643 |
| 1 | 321.47174 | 4.99042 |
| 1 | 321.57174 | 5.60948 |
| 1 | 321.68937 | 6.20686 |
| 1 | 322.48738 | 6.655 |
| 1 | 323.21283 | 7.1838 |
| 1 | 324.7363 | 7.82468 |
| 1 | 325.96957 | 8.85487 |
| 1 | 326.98522 | 9.8864 |
| 1 | 327.20285 | 11.01329 |
| 1 | 328.72631 | 12.37949 |
| 1 | 328.81631 | 13.69784 |
| 1 | 328.93631 | 15.50129 |
| 1 | 329.74195 | 16.62049 |
| 1 | 330.7576 | 17.70068 |
| 1 | 331.19287 | 18.97868 |
| 1 | 331.29287 | 21.28507 |
| 1 | 331.70069 | 23.92549 |
| 1 | 331.80069 | 27.32015 |
| 1 | 332.49869 | 36.92784 |
| 1 | 332.49869 | 40.58571 |
| 1 | 331.99087 | 46.97375 |
| 1 | 331.70069 | 54.8585 |
| 1 | 331.60069 | 63.63598 |
| 1 | 331.48305 | 71.53003 |
| 1 | 331.48305 | 80.22274 |
| 1 | 330.7576 | 93.68848 |
| 1 | 329.74195 | 110.40316 |
| 1 | 329.23413 | 126.92106 |
| 1 | 329.13413 | 142.66565 |
| 1 | 329.10413 | 164.01048 |
| 1 | 328.94395 | 191.54037 |
| 1 | 327.71067 | 233.98176 |
| 1 | 327.91067 | 280.10048 |
| 1 | 328.50868 | 309.92957 |
| 1 | 328.00086 | 329.33092 |
| 1 | 328.20086 | 376.05793 |
| 1 | 328.40086 | 418.92267 |
| 1 | 328.72631 | 456.29582 |
| 1 | 328.94395 | 504.88871 |
| 1 | 328.84395 | 558.65645 |
| 1 | 328.64395 | 622.33458 |
| 1 | 328.21849 | 693.27101 |
| 1 | 328.31849 | 835.53536 |
| 1 | 328.42849 | 1013.81001 |
| 1 | 328.45849 | 1221.85134 |
| 1 | 328.63849 | 1351.9715 |
| 1 | 328.64849 | 1655.25865 |
| 1 | 328.72631 | 1963.77228 |
| 1 | 330.24978 | 2187.61169 |
| 1 | 330.14978 | 2458.98533 |
| 1 | 329.45177 | 3003.8457 |
| 1 | 326.69503 | 3484.46733 |
| 1 | 326.69503 | 4363.16071 |
| 1 | 327.20285 | 5176.38378 |
| 1 | 327.30285 | 6629.14871 |
| 1 | 327.40285 | 8061.69102 |
| 1 | 320.4561 | 9184.84307 |
| 1 | 311.17024 | 9123.08657 |
| 1 | 299.708 | 9629.00364 |
| 1 | 282.15193 | 9478.61349 |
| 1 | 230.64442 | 9330.57219 |
| 1.2 | 331.70069 | 1.09125 |
| 1.2 | 333.22415 | 1.57087 |
| 1.2 | 335.47307 | 1.95378 |
| 1.2 | 336.48871 | 2.38669 |
| 1.2 | 339.97091 | 3.03597 |
| 1.2 | 339.99091 | 3.57761 |
| 1.2 | 341.49437 | 4.47979 |
| 1.2 | 342.21983 | 5.1849 |
| 1.2 | 344.46875 | 5.97405 |
| 1.2 | 346.50003 | 7.02405 |
| 1.2 | 348.24113 | 8.2586 |
| 1.2 | 348.53131 | 10.67196 |
| 1.2 | 348.02349 | 11.80846 |
| 1.2 | 347.51567 | 14.32798 |
| 1.2 | 349.25677 | 15.99708 |
| 1.2 | 350.78023 | 17.94113 |
| 1.2 | 350.27241 | 22.31437 |
| 1.2 | 351.2155 | 26.23635 |
| 1.2 | 351.50569 | 30.91709 |
| 1.2 | 351.42569 | 38.45329 |
| 1.2 | 350.27241 | 42.16733 |
| 1.2 | 349.25677 | 48.15023 |
| 1.2 | 349.15677 | 53.15827 |
| 1.2 | 349.12677 | 58.42384 |
| 1.2 | 348.96659 | 64.64564 |
| 1.2 | 348.98959 | 72.50172 |
| 1.2 | 349.47441 | 82.7886 |
| 1.2 | 349.25677 | 91.60512 |
| 1.2 | 349.25677 | 105.31056 |
| 1.2 | 350.27241 | 120.25251 |
| 1.2 | 350.17241 | 141.38809 |
| 1.2 | 348.74895 | 164.01048 |
| 1.2 | 349.25677 | 199.0047 |
| 1.2 | 350.49005 | 218.2257 |
| 1.2 | 350.99787 | 275.72574 |
| 1.2 | 351.2155 | 316.97827 |
| 1.2 | 352.01351 | 356.29949 |
| 1.2 | 353.97224 | 432.3216 |
| 1.2 | 353.87224 | 481.59952 |
| 1.2 | 351.50569 | 580.42731 |
| 1.2 | 351.40569 | 693.27101 |
| 1.2 | 350.78023 | 910.07559 |
| 1.2 | 351.2155 | 1022.97064 |
| 1.2 | 351.50569 | 1221.85134 |
| 1.2 | 349.25677 | 1426.94453 |
| 1.2 | 348.02349 | 1578.90593 |
| 1.2 | 349.25677 | 1831.5342 |
| 1.2 | 351.50569 | 2119.8111 |
| 1.2 | 351.2155 | 2308.92481 |
| 1.2 | 353.75461 | 3301.39008 |
| 1.2 | 353.95461 | 3855.54311 |
| 1.2 | 354.77025 | 4827.81229 |
| 1.2 | 345.77457 | 5095.53666 |
| 1.2 | 330.7576 | 5176.38378 |
| 1.2 | 307.68804 | 5211.42404 |
