# Supplementary material for: Prediction of 316 stainless steel low-cycle fatigue life based on machine learning
Source: Sci Rep. 2023 Apr 25;13:6753. doi: 10.1038/s41598-023-33354-1 (PMC10130168; doi:10.1038/s41598-023-33354-1)
Supplement: Supplementary file 2 — Supplementary Information 2. [file 41598_2023_33354_MOESM2_ESM.docx]

**Table 1. original data**

|  | Crack growth rate, mm/N | Strain amplitude, % | Residual stress, MPa | Fatigue life, N |
| --- | --- | --- | --- | --- |
| 1 | 2.8×10^-6^ | 0.3796 | -306.4 | 314 |
| 2 | 3.9×10^-6^ | 0.1584 | -294.8 | 424 |
| 3 | 4.9×10^-6^ | 0.2375 | -299.3 | 622 |
| 4 | 6.7×10^-6^ | 0.3408 | -339.9 | 664 |
| 5 | 8.9×10^-6^ | 0.2575 | -312.5 | 586 |
| 6 | 9.7×10^-6^ | 0.2222 | -257.8 | 6404 |
| 7 | 2.8×10^-6^ | 0.2864 | -364.6 | 4664 |
| 8 | 1.1×10^-5^ | 0.2713 | -392.1 | 26524 |
| 9 | 1.2×10^-5^ | 0.5435 | -385.6 | 17204 |
| 10 | 1.6×10^-5^ | 0.7888 | -347.8 | 23864 |
| 11 | 0.1×10^-6^ | 0.5781 | -370 | 90497 |
| 12 | 0.1×10^-6^ | 0.3906 | -552.4 | 90108 |
| 13 | 0.1×10^-6^ | 0.1563 | -396.6 | 89112 |
| 14 | 0.1×10^-6^ | 0.2589 | -557.2 | 91120 |
| 15 | 0.1×10^-6^ | 0.403 | -457.9 | 89511 |
| 16 | 0.1×10^-6^ | 0.8529 | -428 | 88105 |
| 17 | 0.1×10^-6^ | 0.3028 | -409.7 | 90653 |
| 18 | 0.1×10^-6^ | 0.5959 | -685.9 | 87128 |
| 19 | 0.1×10^-6^ | 0.25 | -1326 | 84251 |
| 20 | 0.1×10^-6^ | 0.2655 | -1130 | 85957 |
| 21 | 0.1×10^-6^ | 0.3713 | -1293 | 90108 |
| 22 | 0.1×10^-6^ | 0.2025 | -556.7 | 91569 |
| 23 | 0.1×10^-6^ | 1.547 | -201.9 | 85713 |
| 24 | 0.1×10^-6^ | 0.9806 | -603.4 | 88249 |
| 25 | 0.1×10^-6^ | 0.8191 | -290.2 | 86403 |
| 26 | 0.1×10^-6^ | 0.5115 | -1094 | 85278 |
| 27 | 0.7×10^-6^ | 0.4375 | -673.7 | 90846 |
| 28 | 0.7×10^-6^ | 0.1814 | -769 | 88622 |
| 29 | 0.7×10^-6^ | 0.4331 | -585 | 86993 |
| 30 | 0.7×10^-6^ | 0.5204 | -506.3 | 86109 |
| 31 | 0.7×10^-6^ | 0.9948 | -420.5 | 91351 |
| 32 | 0.7×10^-6^ | 0.4057 | -512.2 | 88462 |
| 33 | 0.7×10^-6^ | 0.8601 | -2501 | 91129 |
| 34 | 0.7×10^-6^ | 0.6422 | -806 | 86106 |
| 35 | 0.7×10^-6^ | 0.4537 | -810 | 90844 |
| 36 | 0.7×10^-6^ | 0.1767 | -651.9 | 86159 |
| 37 | 1×10^-6^ | 0.7456 | -813.7 | 89539 |
| 38 | 1×10^-6^ | 0.4697 | -514.3 | 89760 |
| 39 | 1×10^-6^ | 0.23 | -950 | 9081 |
| 40 | 1×10^-6^ | 0.7311 | -758.6 | 8996 |
| 41 | 1×10^-6^ | 0.3105 | -1010 | 8999 |
| 42 | 1×10^-6^ | 0.2684 | -551.7 | 8618 |
| 43 | 1×10^-6^ | 0.5907 | -1290 | 88104 |
| 44 | 1×10^-6^ | 0.338 | -1092 | 89763 |
| 45 | 1×10^-6^ | 0.3628 | -991.7 | 90524 |
| 46 | 1×10^-6^ | 0.6107 | -467.8 | 90791 |
| 47 | 1.2×10^-6^ | 0.5506 | -1203 | 84300 |
| 48 | 1.2×10^-6^ | 0.6592 | -565.4 | 87163 |
| 49 | 1.2×10^-6^ | 0.5462 | -633.1 | 89221 |
| 50 | 1.2×10^-6^ | 0.2512 | -507.4 | 87509 |
| 51 | 1.2×10^-6^ | 0.4709 | -712.8 | 85743 |
| 52 | 1.2×10^-6^ | 0.3147 | -514 | 89686 |
| 53 | 1.2×10^-6^ | 1.095 | -929.4 | 85971 |
| 54 | 1.2×10^-6^ | 0.2271 | -321.6 | 89867 |
| 55 | 1.2×10^-6^ | 0.3908 | -629.9 | 86409 |
| 56 | 1.2×10^-6^ | 0.7049 | -1174 | 90312 |
| 57 | 0.7×10^-6^ | 0.5158 | -982 | 86512 |
| 58 | 0.7×10^-6^ | 0.334 | -713.3 | 88677 |
| 59 | 0.7×10^-6^ | 0.2467 | -656.1 | 90656 |
| 60 | 0.7×10^-6^ | 0.2451 | -417.2 | 89306 |
| 61 | 0.7×10^-6^ | 0.4226 | -1145 | 8912 |
| 62 | 0.7×10^-6^ | 0.6412 | -815.8 | 91191 |
| 63 | 0.7×10^-6^ | 0.2121 | -1001 | 84230 |
| 64 | 0.7×10^-6^ | 0.6137 | -443.3 | 86120 |
| 65 | 0.7×10^-6^ | 0.9811 | -530.2 | 8630 |
| 66 | 0.7×10^-6^ | 0.4347 | -813 | 8691 |
| 67 | 1×10^-6^ | 0.236 | -311.9 | 8589 |
| 68 | 1×10^-6^ | 0.2241 | -807.2 | 854 |
| 69 | 1×10^-6^ | 0.3534 | -313.1 | 8812 |
| 70 | 1×10^-6^ | 0.8307 | -420.3 | 8743 |
| 71 | 1×10^-6^ | 0.3833 | -656.9 | 8577 |
| 72 | 1×10^-6^ | 0.2244 | -435.6 | 89111 |
| 73 | 1×10^-6^ | 0.2142 | -578.3 | 84667 |
| 74 | 1×10^-6^ | 0.1559 | -1335 | 9012 |
| 75 | 1×10^-6^ | 0.1967 | -1104 | 8556 |
| 76 | 1×10^-6^ | 0.4993 | -590 | 88411 |
| 77 | 1.2×10^-6^ | 0.1199 | -464.1 | 87139 |
| 78 | 1.2×10^-6^ | 0.6362 | -143.5 | 8627 |
| 79 | 1.2×10^-6^ | 0.4302 | -421 | 8991 |
| 80 | 1.2×10^-6^ | 0.4565 | -1088 | 8534 |
| 81 | 1.2×10^-6^ | 0.3117 | -469.1 | 9049 |
| 82 | 1.2×10^-6^ | 0.51 | -575.3 | 9068 |
| 83 | 1.2×10^-6^ | 0.2351 | -606.5 | 9094 |
| 84 | 1.2×10^-6^ | 0.3037 | -224.5 | 8584 |
| 85 | 1.2×10^-6^ | 0.4062 | -373.2 | 9055 |
| 86 | 1.2×10^-6^ | 1.509 | -514.5 | 9051 |
| 87 | 0.3×10^-6^ | 0.1913 | -466.7 | 8632 |
| 88 | 0.3×10^-6^ | 0.3344 | -1024 | 8771 |
| 89 | 0.3×10^-6^ | 1.215 | -271.2 | 9059 |
| 90 | 0.3×10^-6^ | 0.3776 | -582.7 | 8627 |
| 91 | 0.3×10^-6^ | 0.2623 | -761.7 | 8673 |
| 92 | 0.3×10^-6^ | 0.2136 | -928.8 | 9094 |
| 93 | 0.3×10^-6^ | 0.6003 | -269.4 | 8594 |
| 94 | 0.3×10^-6^ | 0.5381 | -832.9 | 87281 |
| 95 | 0.3×10^-6^ | 0.1916 | -427.9 | 8573 |
| 96 | 0.3×10^-6^ | 0.3563 | -1761 | 8654 |
| 97 | 0.3×10^-6^ | 0.2527 | -329.6 | 88147 |
| 98 | 0.3×10^-6^ | 0.5959 | -402 | 873 |
| 99 | 0.3×10^-6^ | 1.2873 | -1509 | 863 |
| 100 | 0.3×10^-6^ | 0.2619 | -289.1 | 9113 |
| 101 | 0.3×10^-6^ | 0.3977 | -441.3 | 9110 |
| 102 | 0.3×10^-6^ | 0.2747 | -558.1 | 90401 |
| 103 | 0.3×10^-6^ | 0.2563 | -955.1 | 853 |
| 104 | 0.3×10^-6^ | 0.3677 | -241 | 9010 |
| 105 | 0.3×10^-6^ | 0.4202 | -399.8 | 905 |
| 106 | 0.3×10^-6^ | 0.3354 | -585.9 | 90944 |
| 107 | 1.5×10^-6^ | 0.1408 | -458.4 | 9011 |
| 108 | 1.5×10^-6^ | 0.3371 | -1075 | 867 |
| 109 | 1.5×10^-6^ | 0.4309 | -1099 | 8810 |
| 110 | 1.5×10^-6^ | 0.1859 | -310.8 | 897 |
| 111 | 1.5×10^-6^ | 0.4209 | -1288 | 887 |
| 112 | 1.5×10^-6^ | 0.1851 | -433.8 | 905 |
| 113 | 1.5×10^-6^ | 0.2796 | -578.9 | 8810 |
| 114 | 1.5×10^-6^ | 0.2787 | -324.2 | 894 |
| 115 | 1.5×10^-6^ | 0.2005 | -519.4 | 87100 |
| 116 | 1.5×10^-6^ | 1.088 | -623.9 | 883 |
| 117 | 1.5×10^-6^ | 0.2456 | -403.3 | 865 |
| 118 | 1.5×10^-6^ | 0.163 | -793.2 | 896 |
| 119 | 1.5×10^-6^ | 0.5659 | -674.5 | 866 |
| 120 | 1.5×10^-6^ | 0.3665 | -428.9 | 874 |
| 121 | 1.5×10^-6^ | 0.3971 | -736.9 | 871 |
| 122 | 1.5×10^-6^ | 0.3198 | -307.3 | 907 |
| 123 | 1.5×10^-6^ | 0.2727 | -671.4 | 87556 |
| 124 | 1.5×10^-6^ | 0.253 | -584.1 | 859 |
| 125 | 1.5×10^-6^ | 0.2913 | -455.8 | 866 |
| 126 | 1.5×10^-6^ | 0.404 | -984.6 | 8712 |
| 127 | 0.7×10^-6^ | 0.5296 | -408.2 | 906 |
| 128 | 0.7×10^-6^ | 0.3132 | -747.2 | 912 |
| 129 | 0.7×10^-6^ | 0.6896 | -1167 | 913 |
| 130 | 0.7×10^-6^ | 0.1504 | -509.2 | 908 |
| 131 | 0.7×10^-6^ | 0.2543 | -951.6 | 877 |
| 132 | 0.7×10^-6^ | 0.4101 | -344.9 | 8910 |
| 133 | 0.7×10^-6^ | 0.422 | -394.1 | 859 |
| 134 | 0.7×10^-6^ | 0.3251 | -685 | 907 |
| 135 | 0.7×10^-6^ | 0.355 | -516.6 | 8912 |
| 136 | 0.7×10^-6^ | 0.3197 | -527.2 | 857 |
| 137 | 1×10^-6^ | 0.4212 | -948 | 58109 |
| 138 | 1×10^-6^ | 0.5925 | -245.2 | 864 |
| 139 | 1×10^-6^ | 0.2385 | -1138 | 866 |
| 140 | 1×10^-6^ | 0.7036 | -609.1 | 903 |
| 141 | 1×10^-6^ | 0.1707 | -593.7 | 901 |
| 142 | 1×10^-6^ | 0.2562 | -559.2 | 8610 |
| 143 | 1×10^-6^ | 0.2986 | -689.5 | 56973 |
| 144 | 1×10^-6^ | 0.1482 | -401.5 | 861 |
| 145 | 1×10^-6^ | 0.1728 | -336.1 | 862 |
| 146 | 1×10^-6^ | 0.2208 | -432 | 869 |
| 147 | 1.2×10^-6^ | 0.1194 | -998.9 | 8911 |
| 148 | 1.2×10^-6^ | 0.1833 | -1274 | 894 |
| 149 | 1.2×10^-6^ | 0.9291 | -563 | 855 |
| 150 | 1.2×10^-6^ | 0.9317 | -645.7 | 858 |
| 151 | 1.2×10^-6^ | 0.2742 | -402.7 | 898 |
| 152 | 1.2×10^-6^ | 0.3316 | -386.3 | 901 |
| 153 | 1.2×10^-6^ | 0.2479 | -990 | 9110 |
| 154 | 1.2×10^-6^ | 0.1588 | -358.9 | 911 |
| 155 | 1.2×10^-6^ | 1.292 | -357.6 | 87 |
| 156 | 1.2×10^-6^ | 0.1816 | -599.5 | 908 |
| 157 | 0.2×10^-6^ | 1.008 | -447.8 | 90 |
| 158 | 0.2×10^-6^ | 0.2114 | -541.8 | 902 |
| 159 | 0.2×10^-6^ | 0.295 | -930.9 | 61129 |
| 160 | 0.2×10^-6^ | 0.1783 | -1245 | 8610 |
| 161 | 0.2×10^-6^ | 0.2684 | -441 | 8912 |
| 162 | 0.2×10^-6^ | 0.2569 | -602.9 | 91 |
| 163 | 0.2×10^-6^ | 0.6534 | -587.4 | 8911 |
| 164 | 0.2×10^-6^ | 0.1844 | -546.3 | 912 |
| 165 | 0.2×10^-6^ | 0.2054 | -773.5 | 887 |
| 166 | 0.2×10^-6^ | 0.2382 | -412.7 | 71132 |
| 167 | 0.3×10^-6^ | 0.3424 | -1482 | 9012 |
| 168 | 0.3×10^-6^ | 0.9553 | -361.6 | 9013 |
| 169 | 0.3×10^-6^ | 0.3265 | -555.1 | 862 |
| 170 | 0.3×10^-6^ | 0.6061 | -711.8 | 9012 |
| 171 | 0.3×10^-6^ | 0.599 | -289.9 | 8910 |
| 172 | 0.3×10^-6^ | 0.2144 | -561.3 | 8812 |
| 173 | 0.3×10^-6^ | 0.2345 | -716.6 | 9010 |
| 174 | 0.3×10^-6^ | 0.3661 | -280.5 | 898 |
| 175 | 0.3×10^-6^ | 0.3368 | -710.6 | 873 |
| 176 | 0.3×10^-6^ | 0.4266 | -463.7 | 874 |
| 177 | 0.4×10^-6^ | 0.3699 | -480.4 | 86 |
| 178 | 0.4×10^-6^ | 0.7548 | -1230 | 8811 |
| 179 | 0.4×10^-6^ | 0.647 | -504.1 | 29382 |
| 180 | 0.4×10^-6^ | 0.3414 | -449.9 | 9113 |
| 181 | 0.4×10^-6^ | 0.2841 | -886.3 | 8712 |
| 182 | 0.4×10^-6^ | 0.2387 | -390 | 897 |
| 183 | 0.4×10^-6^ | 0.1601 | -512.2 | 8910 |
| 184 | 0.4×10^-6^ | 0.425 | -451.1 | 86 |
| 185 | 0.4×10^-6^ | 1.072 | -221.2 | 894 |
| 186 | 0.4×10^-6^ | 0.4222 | -682.5 | 864 |
| 187 | 0.6×10^-6^ | 0.3639 | -766.6 | 873 |
| 188 | 0.6×10^-6^ | 0.3309 | -1007 | 914 |
| 189 | 0.6×10^-6^ | 0.2368 | -744.7 | 903 |
| 190 | 0.6×10^-6^ | 0.21 | -1386 | 9012 |
| 191 | 0.6×10^-6^ | 0.1911 | -446.2 | 903 |
| 192 | 0.6×10^-6^ | 0.6997 | -800 | 905 |
| 193 | 0.6×10^-6^ | 0.3135 | -418.7 | 893 |
| 194 | 0.6×10^-6^ | 0.163 | -602.4 | 886 |
| 195 | 0.6×10^-6^ | 0.4653 | -1247 | 49263 |
| 196 | 0.6×10^-6^ | 0.4953 | -384.6 | 901 |
| 197 | 0.8×10^-6^ | 0.306 | -508.3 | 70401 |
| 198 | 0.8×10^-6^ | 1.207 | -349.6 | 871 |
| 199 | 0.8×10^-6^ | 0.2204 | -502.5 | 859 |
| 200 | 0.8×10^-6^ | 0.2446 | -489.9 | 69511 |
| 201 | 0.8×10^-6^ | 0.2505 | -321.6 | 90317 |
| 202 | 0.8×10^-6^ | 0.2868 | -1075 | 8612 |
| 203 | 0.8×10^-6^ | 0.4157 | -415.1 | 9112 |
| 204 | 0.8×10^-6^ | 0.2428 | -541.6 | 884 |
| 205 | 0.8×10^-6^ | 0.5495 | -244.5 | 894 |
| 206 | 0.8×10^-6^ | 0.2431 | -1192 | 866 |
| 207 | 0.2×10^-6^ | 0.3428 | -644.2 | 911 |
| 208 | 0.3×10^-6^ | 0.6917 | -846 | 9011 |
| 209 | 0.4×10^-6^ | 0.3446 | -674.8 | 9110 |
| 210 | 0.6×10^-6^ | 0.258 | -686.9 | 911 |
| 211 | 0.8×10^-6^ | 0.2871 | -470.9 | 8610 |
| 212 | 0.2×10^-6^ | 0.2298 | -334.2 | 892 |
| 213 | 0.2×10^-6^ | 0.1153 | -664.7 | 90 |
| 214 | 0.2×10^-6^ | 0.5243 | -389.4 | 883 |
| 215 | 0.2×10^-6^ | 0.3921 | -1076 | 76135 |
| 216 | 0.2×10^-6^ | 0.1781 | -571.1 | 912 |
| 217 | 0.2×10^-6^ | 0.6874 | -668.7 | 89 |
| 218 | 0.3×10^-6^ | 0.3478 | -1104 | 851 |
| 219 | 0.3×10^-6^ | 0.4312 | -420.3 | 49864 |
| 220 | 0.3×10^-6^ | 0.3975 | -572.3 | 8911 |
| 221 | 0.3×10^-6^ | 0.3719 | -534.6 | 85 |
| 222 | 0.3×10^-6^ | 0.4207 | -758.6 | 889 |
| 223 | 0.3×10^-6^ | 0.5366 | -476.5 | 883 |
| 224 | 0.3×10^-6^ | 0.2954 | -1155 | 884 |
| 225 | 0.4×10^-6^ | 1.214 | -476.7 | 913 |
| 226 | 0.4×10^-6^ | 0.2699 | -290.9 | 9112 |
| 227 | 0.4×10^-6^ | 0.2073 | -1206 | 883 |
| 228 | 0.4×10^-6^ | 0.1115 | -618.4 | 911 |
| 229 | 0.4×10^-6^ | 0.4751 | -840.4 | 9012 |
| 230 | 0.4×10^-6^ | 0.3276 | -530.6 | 893 |
| 231 | 0.4×10^-6^ | 0.4866 | -337.7 | 884 |
| 232 | 0.6×10^-6^ | 0.7474 | -610.7 | 8810 |
| 233 | 0.6×10^-6^ | 0.184 | -920.6 | 38330 |
| 234 | 0.6×10^-6^ | 0.5169 | -501.3 | 40769 |
| 235 | 0.6×10^-6^ | 0.2889 | -698.8 | 855 |
| 236 | 0.6×10^-6^ | 0.9761 | -566.3 | 8510 |
| 237 | 0.6×10^-6^ | 0.2194 | -372.7 | 871 |
| 238 | 0.6×10^-6^ | 0.335 | -338.3 | 901 |
| 239 | 0.6×10^-6^ | 0.5018 | -595.9 | 91 |
| 240 | 0.7×10^-6^ | 0.2344 | -333.6 | 907 |
| 241 | 0.7×10^-6^ | 0.281 | -394.1 | 8913 |
| 242 | 0.7×10^-6^ | 0.2152 | -817.7 | 8610 |
| 243 | 0.7×10^-6^ | 0.1485 | -690.2 | 8712 |
| 244 | 0.7×10^-6^ | 0.1312 | -234.3 | 903 |
| 245 | 0.7×10^-6^ | 0.286 | -508.8 | 873 |
| 246 | 0.7×10^-6^ | 0.2574 | -1234 | 9011 |
| 247 | 0.2×10^-6^ | 0.2545 | -455.3 | 912 |
| 248 | 0.2×10^-6^ | 0.3491 | -294.5 | 862 |
| 249 | 0.2×10^-6^ | 0.1415 | -419.8 | 8711 |
| 250 | 0.2×10^-6^ | 0.6009 | -396.5 | 864 |
| 251 | 0.2×10^-6^ | 0.4674 | -542.9 | 9113 |
| 252 | 0.2×10^-6^ | 0.2185 | -279.6 | 882 |
| 253 | 0.2×10^-6^ | 0.3778 | -334.3 | 892 |
| 254 | 0.3×10^-6^ | 0.1903 | -409.1 | 893 |
| 255 | 0.3×10^-6^ | 0.4834 | -609.9 | 874 |
| 256 | 0.3×10^-6^ | 0.8113 | -371.5 | 909 |
| 257 | 0.3×10^-6^ | 0.1499 | -644.8 | 852 |
| 258 | 0.3×10^-6^ | 0.1705 | -641.2 | 89 |
| 259 | 0.3×10^-6^ | 0.522 | -381.1 | 60769 |
| 260 | 0.3×10^-6^ | 0.4007 | -899.3 | 75382 |
| 261 | 0.4×10^-6^ | 0.2251 | -1191 | 9111 |
| 262 | 0.4×10^-6^ | 0.2239 | -588.7 | 86 |
| 263 | 0.4×10^-6^ | 0.2143 | -477.3 | 894 |
| 264 | 0.4×10^-6^ | 0.8361 | -537.3 | 79 |
| 265 | 0.4×10^-6^ | 0.184 | -992.1 | 71376 |
| 266 | 0.4×10^-6^ | 0.2895 | -963.7 | 8711 |
| 267 | 0.4×10^-6^ | 1.291 | -506.9 | 911 |
| 268 | 0.6×10^-6^ | 0.2338 | -579.1 | 9013 |
| 269 | 0.6×10^-6^ | 0.4467 | -641.2 | 68143 |
| 270 | 0.6×10^-6^ | 1.172 | -933.1 | 888 |
| 271 | 0.6×10^-6^ | 0.3642 | -381.9 | 77 |
| 272 | 0.6×10^-6^ | 0.2719 | -466.5 | 51376 |
| 273 | 0.6×10^-6^ | 0.2976 | -512.2 | 869 |
| 274 | 0.7×10^-6^ | 0.1731 | -2250 | 873 |
| 275 | 0.7×10^-6^ | 0.2213 | -464.4 | 897 |
| 276 | 0.7×10^-6^ | 0.2204 | -642.5 | 862 |
| 277 | 0.7×10^-6^ | 0.3961 | -1682 | 915 |
| 278 | 0.7×10^-6^ | 0.7572 | -561 | 857 |
| 279 | 0.7×10^-6^ | 0.6226 | -1040 | 844 |
| 280 | 0.7×10^-6^ | 0.5858 | -1419 | 78995 |
| 281 | 0.4×10^-6^ | 0.207 | -471.3 | 892 |
| 282 | 0.5×10^-6^ | 0.3897 | -489 | 8912 |
| 283 | 0.6×10^-6^ | 0.3704 | -475.9 | 64501 |
| 284 | 0.7×10^-6^ | 0.1458 | -526.4 | 873 |
| 285 | 0.8×10^-6^ | 0.1532 | -496.6 | 9010 |
| 286 | 0.1213×10^-6^ | 0.2273 | -666 | 913 |
| 287 | 0.1259×10^-6^ | 0.3577 | -481.9 | 57 |
| 288 | 0.1172×10^-6^ | 0.3274 | -1297 | 94358 |
| 289 | 0.1256×10^-6^ | 0.1716 | -1194 | 898 |
| 290 | 0.128×10^-6^ | 0.4033 | -904.3 | 879 |
| 291 | 0.1249×10^-6^ | 0.2773 | -432.7 | 891 |
| 292 | 0.1216×10^-6^ | 0.8161 | -981.6 | 9110 |
| 293 | 0.1238×10^-6^ | 0.256 | -545.2 | 856 |
| 294 | 0.1193×10^-6^ | 0.7661 | -507.6 | 90 |
| 295 | 0.1327×10^-6^ | 0.3511 | -515.9 | 89 |
| 296 | 0.1574×10^-6^ | 0.5558 | -437.6 | 857 |
| 297 | 0.1297×10^-6^ | 0.4743 | -668.3 | 859 |
| 298 | 0.1287×10^-6^ | 0.286 | -250.5 | 859 |
| 299 | 0.1467×10^-6^ | 0.1912 | -481.6 | 894 |
| 300 | 0.1338×10^-6^ | 0.2244 | -782.7 | 846 |
| 301 | 0.1218×10^-6^ | 0.4041 | -273.9 | 8510 |
| 302 | 0.1497×10^-6^ | 0.1642 | -918.6 | 69742 |
| 303 | 0.09711×10^-6^ | 0.2191 | -432 | 9111 |
| 304 | 0.1289×10^-6^ | 0.4101 | -1206 | 885 |
| 305 | 0.1526×10^-6^ | 0.3093 | -491.9 | 40103 |
| 306 | 0.1254×10^-6^ | 0.8426 | -1162 | 854 |
| 307 | 0.1357×10^-6^ | 0.4615 | -748.9 | 86 |
| 308 | 0.1478×10^-6^ | 0.1818 | -520 | 8611 |
| 309 | 0.139×10^-6^ | 0.1931 | -442.5 | 9111 |
| 310 | 0.1277×10^-6^ | 0.1988 | -566.2 | 893 |
| 311 | 0.159×10^-6^ | 0.1852 | -1027 | 894 |
| 312 | 0.1142×10^-6^ | 0.4505 | -372.7 | 901 |
| 313 | 0.1665×10^-6^ | 0.6643 | -582.7 | 891 |
| 314 | 0.155×10^-6^ | 0.2963 | -408.8 | 911 |
| 315 | 0.153×10^-6^ | 0.4357 | -928.2 | 8860 |
| 316 | 0.1006×10^-6^ | 0.3129 | -1207 | 88649 |
| 317 | 0.1178×10^-6^ | 0.2084 | -869.5 | 854 |
| 318 | 0.1498×10^-6^ | 0.403 | -340.9 | 909 |
| 319 | 0.1482×10^-6^ | 0.1759 | -529.4 | 8810 |
| 320 | 0.145×10^-6^ | 0.2711 | -288.5 | 868 |
| 321 | 0.1444×10^-6^ | 0.2927 | -520 | 8510 |
| 322 | 0.1401×10^-6^ | 0.3473 | -346.4 | 8910 |
| 323 | 0.09445×10^-6^ | 0.9289 | -1384 | 91115 |
| 324 | 0.09388×10^-6^ | 0.9555 | -432.2 | 8811 |
| 325 | 0.1389×10^-6^ | 0.4053 | -1148 | 877 |
| 326 | 0.1288×10^-6^ | 0.2204 | -728.2 | 861 |
| 327 | 0.1585×10^-6^ | 0.4203 | -537.9 | 9112 |
| 328 | 0.136×10^-6^ | 0.3416 | -994 | 40439 |
| 329 | 0.08949×10^-6^ | 0.7582 | -399.8 | 868 |
| 330 | 0.1251×10^-6^ | 0.1935 | -716.6 | 879 |
| 331 | 0.1072×10^-6^ | 0.5079 | -546.4 | 8813 |
| 332 | 0.1765×10^-6^ | 0.1692 | -761.3 | 8812 |
| 333 | 0.1312×10^-6^ | 0.3962 | -980.5 | 9113 |
| 334 | 0.09545×10^-6^ | 0.3249 | -1123 | 846 |
| 335 | 0.1518×10^-6^ | 0.4489 | -477.4 | 875 |
| 336 | 0.1377×10^-6^ | 0.3118 | -227.2 | 872 |
| 337 | 0.1622×10^-6^ | 0.3721 | -928.3 | 889 |
| 338 | 0.1377×10^-6^ | 0.231 | -286.3 | 9113 |
| 339 | 0.1862×10^-6^ | 0.2949 | -1260 | 849 |
| 340 | 0.1314×10^-6^ | 0.4681 | -203.9 | 87100 |
| 341 | 0.1275×10^-6^ | 0.2959 | -278.6 | 50103 |
| 342 | 0.1446×10^-6^ | 1.046 | -492.9 | 911 |
| 343 | 0.1406×10^-6^ | 1.004 | -432.8 | 869 |
| 344 | 0.1323×10^-6^ | 0.3331 | -409 | 85759 |
| 345 | 0.1368×10^-6^ | 0.3336 | -423.6 | 868 |
| 346 | 0.1359×10^-6^ | 0.524 | -438.6 | 864 |
| 347 | 0.1651×10^-6^ | 0.5706 | -662.7 | 861 |
| 348 | 0.1562×10^-6^ | 1 | -572.6 | 866 |
| 349 | 0.1247×10^-6^ | 0.3342 | -464.5 | 5712 |
| 350 | 0.09368×10^-6^ | 0.2239 | -719.5 | 905 |
| 351 | 0.1385×10^-6^ | 0.4789 | -895 | 881 |
| 352 | 0.1584×10^-6^ | 0.2473 | -912.7 | 852 |
| 353 | 0.09958×10^-6^ | 0.2092 | -1364 | 884 |
| 354 | 0.1491×10^-6^ | 0.2976 | -731.3 | 915 |
| 355 | 0.1048×10^-6^ | 0.1822 | -334.2 | 864 |
| 356 | 0.1147×10^-6^ | 1.058 | -1319 | 68206 |
| 357 | 0.1034×10^-6^ | 0.5904 | -826.8 | 895 |
| 358 | 0.1733×10^-6^ | 0.2102 | -1157 | 8711 |
| 359 | 0.1369×10^-6^ | 0.1839 | -466.1 | 905 |
| 360 | 0.09402×10^-6^ | 0.4455 | -386.8 | 88203 |
| 361 | 0.1184×10^-6^ | 0.1938 | -880.2 | 8711 |
| 362 | 0.08822×10^-6^ | 0.2525 | -857.6 | 913 |
| 363 | 0.1526×10^-6^ | 0.286 | -642.7 | 877 |
| 364 | 0.1265×10^-6^ | 0.3077 | -387.3 | 897 |
| 365 | 0.1223×10^-6^ | 0.1302 | -448.6 | 871 |
| 366 | 0.1436×10^-6^ | 0.7128 | -1670 | 78299 |
| 367 | 0.1548×10^-6^ | 0.338 | -1320 | 91 |
| 368 | 0.1415×10^-6^ | 0.5262 | -568.9 | 8812 |
| 369 | 0.1085×10^-6^ | 0.3539 | -523.8 | 854 |
| 370 | 0.1696×10^-6^ | 0.645 | -465.4 | 9113 |
| 371 | 0.1341×10^-6^ | 0.6289 | -531.5 | 45638 |
| 372 | 0.1121×10^-6^ | 0.2315 | -355.3 | 869 |
| 373 | 0.1701×10^-6^ | 0.2449 | -633 | 854 |
| 374 | 0.1234×10^-6^ | 0.2535 | -359.9 | 90 |
| 375 | 0.1311×10^-6^ | 0.7364 | -632.6 | 8910 |
| 376 | 0.1428×10^-6^ | 0.552 | -1077 | 86 |
| 377 | 0.1255×10^-6^ | 0.37 | -561 | 8910 |
| 378 | 0.1015×10^-6^ | 1.111 | -244 | 859 |
| 379 | 0.1453×10^-6^ | 0.3567 | -288.1 | 875 |
| 380 | 0.1072×10^-6^ | 0.4204 | -431.1 | 892 |
| 381 | 0.1368×10^-6^ | 0.6965 | -1076 | 857 |
| 382 | 0.1465×10^-6^ | 0.2357 | -646.1 | 9113 |
| 383 | 0.1142×10^-6^ | 0.3242 | -496.4 | 38147 |
| 384 | 0.1402×10^-6^ | 0.3927 | -659.7 | 911 |
| 385 | 0.1522×10^-6^ | 0.8973 | -1214 | 871 |
| 386 | 0.1426×10^-6^ | 0.8245 | -782.6 | 864 |
| 387 | 0.146×10^-6^ | 0.2419 | -658.8 | 34799 |
| 388 | 0.1402×10^-6^ | 0.6361 | -1217 | 892 |
| 389 | 0.1297×10^-6^ | 0.7392 | -321.2 | 7811 |
| 390 | 0.1081×10^-6^ | 0.6191 | -648.2 | 56135 |
| 391 | 0.1548×10^-6^ | 0.8337 | -289.7 | 907 |
| 392 | 0.1435×10^-6^ | 0.4332 | -525.2 | 904 |
| 393 | 0.1386×10^-6^ | 0.4255 | -575.3 | 48350 |
| 394 | 0.1356×10^-6^ | 0.361 | -407.4 | 78518 |
| 395 | 0.1531×10^-6^ | 0.2656 | -1878 | 8611 |
| 396 | 0.1126×10^-6^ | 0.121 | -300.2 | 8710 |
| 397 | 0.1316×10^-6^ | 0.5692 | -744.9 | 68725 |
| 398 | 0.1411×10^-6^ | 0.3602 | -1138 | 2912 |
| 399 | 0.1427×10^-6^ | 0.1186 | -1223 | 88119 |
| 400 | 0.1376×10^-6^ | 0.1807 | -588.9 | 875 |
| 401 | 0.1415×10^-6^ | 0.3419 | -1033 | 857 |
| 402 | 0.1541×10^-6^ | 0.1924 | -809.8 | 86730 |
| 403 | 0.1514×10^-6^ | 0.519 | -499 | 85922 |
| 404 | 0.1214×10^-6^ | 0.4388 | -426.7 | 899 |
| 405 | 0.1381×10^-6^ | 0.1745 | -396 | 59143 |
| 406 | 0.1335×10^-6^ | 0.4956 | -395.7 | 58199 |
| 407 | 0.129×10^-6^ | 0.2335 | -798.8 | 54862 |
| 408 | 0.1419×10^-6^ | 0.2571 | -485.8 | 9010 |
| 409 | 0.09926×10^-6^ | 0.2621 | -268.8 | 905 |
| 410 | 0.1021×10^-6^ | 0.22 | -492.1 | 891 |
| 411 | 0.1243×10^-6^ | 0.271 | -592.6 | 909 |
| 412 | 0.1179×10^-6^ | 0.8348 | -458.7 | 862 |
| 413 | 0.1021×10^-6^ | 0.3345 | -1132 | 8611 |
| 414 | 0.1066×10^-6^ | 0.2367 | -704.4 | 8511 |
| 415 | 0.1153×10^-6^ | 0.2621 | -788.5 | 901 |
| 416 | 0.1451×10^-6^ | 0.2877 | -386.1 | 24348 |
| 417 | 0.0997×10^-6^ | 0.6642 | -446 | 895 |
| 418 | 0.1557×10^-6^ | 0.1665 | -616.5 | 89 |
| 419 | 0.1219×10^-6^ | 0.2577 | -520.2 | 4112 |
| 420 | 0.1385×10^-6^ | 0.3278 | -460.3 | 8711 |
| 421 | 0.1315×10^-6^ | 0.3582 | -538.9 | 8711 |
| 422 | 0.1054×10^-6^ | 0.1639 | -947.8 | 888 |
| 423 | 0.1094×10^-6^ | 0.3061 | -477.1 | 843 |
| 424 | 0.164×10^-6^ | 0.4311 | -506.3 | 868 |
| 425 | 0.1531×10^-6^ | 1.37 | -546.1 | 901 |
| 426 | 0.161×10^-6^ | 0.2843 | -687.3 | 89 |
| 427 | 0.1263×10^-6^ | 0.2865 | -1686 | 3712 |
| 428 | 0.1332×10^-6^ | 0.1601 | -230.9 | 864 |
| 429 | 0.1436×10^-6^ | 0.2315 | -1041 | 874 |
| 430 | 0.1613×10^-6^ | 0.3795 | -403.1 | 89 |
| 431 | 0.08799×10^-6^ | 0.1753 | -221.8 | 858 |
| 432 | 0.1166×10^-6^ | 0.4727 | -553.5 | 865 |
| 433 | 0.16×10^-6^ | 0.1942 | -503.2 | 881 |
| 434 | 0.1639×10^-6^ | 0.2023 | -302.4 | 872 |
| 435 | 0.1116×10^-6^ | 0.7275 | -803.1 | 20602 |
| 436 | 0.1064×10^-6^ | 0.5619 | -398 | 869 |
| 437 | 0.1185×10^-6^ | 0.1555 | -380.3 | 906 |
| 438 | 0.1393×10^-6^ | 0.5648 | -373.9 | 89 |
| 439 | 0.1504×10^-6^ | 0.2253 | -412.6 | 762 |
| 440 | 0.1342×10^-6^ | 0.3438 | -797.8 | 745 |
| 441 | 0.1294×10^-6^ | 0.2522 | -363.7 | 791 |
| 442 | 0.2184×10^-6^ | 0.2666 | -684.5 | 748 |
| 443 | 0.1339×10^-6^ | 0.2497 | -516.4 | 875 |
| 444 | 0.1126×10^-6^ | 0.2366 | -537.3 | 51132 |
| 445 | 0.1467×10^-6^ | 0.3186 | -1152 | 857 |
| 446 | 0.1376×10^-6^ | 0.243 | -427.3 | 8912 |
| 447 | 0.166×10^-6^ | 0.316 | -599.4 | 911 |
| 448 | 0.1233×10^-6^ | 0.5058 | -597.8 | 87 |
| 449 | 0.1301×10^-6^ | 0.8068 | -495 | 906 |
| 450 | 0.1096×10^-6^ | 0.3063 | -431.9 | 904 |
| 451 | 0.1287×10^-6^ | 0.3389 | -442.7 | 904 |
| 452 | 0.1353×10^-6^ | 0.2137 | -462 | 902 |
| 453 | 0.1194×10^-6^ | 0.7576 | -347 | 38466 |
| 454 | 0.1041×10^-6^ | 0.2212 | -371.1 | 855 |
| 455 | 0.1168×10^-6^ | 0.2094 | -693.7 | 905 |
| 456 | 0.1166×10^-6^ | 1.296 | -445.3 | 898 |
| 457 | 0.1256×10^-6^ | 0.1904 | -664.9 | 39382 |
| 458 | 0.114×10^-6^ | 0.2134 | -781 | 44610 |
| 459 | 0.1347×10^-6^ | 0.3892 | -904.6 | 852 |
| 460 | 0.1878×10^-6^ | 0.3384 | -519.8 | 844 |
| 461 | 0.1414×10^-6^ | 0.386 | -838.1 | 9010 |
| 462 | 0.1309×10^-6^ | 0.2873 | -295.4 | 879 |
| 463 | 0.1681×10^-6^ | 0.5702 | -1264 | 86 |
| 464 | 0.1192×10^-6^ | 0.4825 | -504.8 | 894 |
| 465 | 0.1432×10^-6^ | 0.3305 | -513.7 | 891 |
| 466 | 0.1206×10^-6^ | 0.1803 | -678.1 | 868 |
| 467 | 0.1223×10^-6^ | 0.2034 | -388 | 8910 |
| 468 | 0.1512×10^-6^ | 0.5835 | -725.5 | 881 |
| 469 | 0.1347×10^-6^ | 0.3106 | -611.2 | 909 |
| 470 | 0.08864×10^-6^ | 0.1402 | -384.8 | 868 |
| 471 | 0.09534×10^-6^ | 0.2636 | -701.9 | 61594 |
| 472 | 0.1076×10^-6^ | 0.2113 | -805.1 | 8912 |
| 473 | 0.1296×10^-6^ | 0.2608 | -1264 | 895 |
| 474 | 0.1384×10^-6^ | 0.2406 | -440.6 | 853 |
| 475 | 0.1343×10^-6^ | 0.5539 | -392 | 903 |
| 476 | 0.1398×10^-6^ | 0.2818 | -298.3 | 862 |
| 477 | 0.1406×10^-6^ | 0.6242 | -404.9 | 911 |
| 478 | 0.1101×10^-6^ | 0.7923 | -577.9 | 74458 |
| 479 | 0.1221×10^-6^ | 0.2929 | -512 | 9010 |
| 480 | 0.1275×10^-6^ | 0.3283 | -580.6 | 9013 |
| 481 | 0.1347×10^-6^ | 0.3534 | -449.3 | 857 |
| 482 | 0.1363×10^-6^ | 0.8811 | -461.4 | 794 |
| 483 | 0.1226×10^-6^ | 0.1689 | -462.9 | 90 |
| 484 | 0.1065×10^-6^ | 0.4768 | -640.1 | 8697 |
| 485 | 0.1176×10^-6^ | 0.1872 | -1685 | 878 |
| 486 | 0.1143×10^-6^ | 1.009 | -366.8 | 471 |
| 487 | 0.1312×10^-6^ | 0.306 | -1407 | 803 |
| 488 | 0.1347×10^-6^ | 0.213 | -1068 | 914 |
| 489 | 0.1249×10^-6^ | 1.167 | -640.7 | 865 |
| 490 | 0.1199×10^-6^ | 0.2541 | -1102 | 8911 |
| 491 | 1.9×10^-5^ | 0.1746 | -328.4 | 34 |
| 492 | 2.2×10^-5^ | 0.439 | -317.8 | 55 |
| 493 | 2.1×10^-5^ | 0.2318 | -232.2 | 57 |
| 494 | 2.3×10^-5^ | 0.4245 | -220.7 | 89 |
| 495 | 2.4×10^-5^ | 0.1855 | -293.1 | 93 |
| 496 | 2.1×10^-5^ | 0.3152 | -385.8 | 124 |
| 497 | 3.5×10^-5^ | 0.2562 | -125.4 | 83 |
| 498 | 1.2×10^-4^ | 0.4098 | -328.4 | 210 |
| 499 | 1.5×10^-4^ | 0.2978 | -353.1 | 267 |
| 500 | 2.1×10^-4^ | 0.6298 | -138.5 | 502 |
